# Loss of *slc39a14* causes simultaneous manganese hypersensitivity and deficiency in zebrafish

**DOI:** 10.1242/dmm.044594

**Published:** 2022-06-15

**Authors:** Karin Tuschl, Richard J. White, Chintan Trivedi, Leonardo E. Valdivia, Stephanie Niklaus, Isaac H. Bianco, Chris Dadswell, Ramón González-Méndez, Ian M. Sealy, Stephan C. F. Neuhauss, Corinne Houart, Jason Rihel, Stephen W. Wilson, Elisabeth M. Busch-Nentwich

**Affiliations:** 1 UCL GOS Institute of Child Health, University College London, 30 Guilford Street, London WC1N 1EH, UK; 2 Department of Cell and Developmental Biology, University College London, Gower Street, London WC1E 6BT, UK; 3 Department of Developmental Neurobiology and MRC Centre for Neurodevelopmental Disorders, IoPPN, Kings College London, New Hunt's House, Guy's Campus, London SE1 1UL, UK; 4 School of Biological and Behavioural Sciences, Faculty of Science and Engineering, Queen Mary University of London, London E1 4NS, UK; 5 Cambridge Institute of Therapeutic Immunology & Infectious Disease (CITIID), Jeffrey Cheah Biomedical Centre, University of Cambridge, Puddicombe Way, Cambridge CB2 0AW, UK; 6 Center for Integrative Biology, Facultad de Ciencias, Universidad Mayor, Camino La Pirámide 5750, Huechuraba 8580745, Chile; 7 Escuela de Biotecnología, Facultad de Ciencias, Universidad Mayor, Camino La Pirámide 5750, Huechuraba 8580745, Chile; 8 Department of Molecular Life Sciences, University of Zurich, Winterthurerstrasse 190, 8057, Zurich, Switzerland; 9 Department of Neuroscience, Physiology & Pharmacology, University College London, Gower Street, London WC1E 6BT, UK; 10 School of Life Sciences, University of Sussex, Brighton BN1 9QJ, UK

**Keywords:** Zebrafish, Slc39a14, Manganese, Calcium, Transcriptome

## Abstract

Manganese neurotoxicity is a hallmark of hypermanganesemia with dystonia 2, an inherited manganese transporter defect caused by mutations in SLC39A14. To identify novel potential targets of manganese neurotoxicity, we performed transcriptome analysis of *slc39a14^−/−^* mutant zebrafish that were exposed to MnCl_2_. Differentially expressed genes mapped to the central nervous system and eye, and pathway analysis suggested that Ca^2+^ dyshomeostasis and activation of the unfolded protein response are key features of manganese neurotoxicity. Consistent with this interpretation, MnCl_2_ exposure led to decreased whole-animal Ca^2+^ levels, locomotor defects and changes in neuronal activity within the telencephalon and optic tectum. In accordance with reduced tectal activity, *slc39a14^−/−^* zebrafish showed changes in visual phototransduction gene expression, absence of visual background adaptation and a diminished optokinetic reflex. Finally, numerous differentially expressed genes in mutant larvae normalised upon MnCl_2_ treatment indicating that, in addition to neurotoxicity, manganese deficiency is present either subcellularly or in specific cells or tissues. Overall, we assembled a comprehensive set of genes that mediate manganese-systemic responses and found a highly correlated and modulated network associated with Ca^2+^ dyshomeostasis and cellular stress.

This article has an associated First Person interview with the first author of the paper.

## Abstract (precis)

**Summary:** Transcriptome analysis of zebrafish *slc39a14^−/−^* mutants demonstrates that loss of *slc39a14* leads to concurrent manganese neurotoxicity and deficiency, both of which are associated with Ca^2+^ dyshomeostasis.

## INTRODUCTION

SLC39A14 is a manganese (Mn) uptake transporter that is essential for the maintenance of Mn homeostasis (Thompson and Wessling-Resnick, 2019). Mutations in SLC39A14 impair cellular Mn uptake and result in systemic Mn overload characterised by hypermanganesemia and neurodegeneration (Tuschl et al., 2016; Juneja et al., 2018; Marti-Sanchez et al., 2018; Rodan et al., 2018; Zeglam et al., 2018). In patients, subsequent accumulation of Mn in the globus pallidus, part of the basal ganglia involved in motor control, leads to rapidly progressive dystonia-parkinsonism with onset in early childhood, a condition known as hypermanganesemia with dystonia 2 (HMNDYT2, OMIM 617013). In a small number of patients, treatment has been attempted using intravenous disodium calcium edetate (Na_2_CaEDTA) for Mn chelation (Tuschl et al., 2016; Rodan et al., 2018; Lee and Shin, 2022), similar to a protocol established for HMNDYT1 (OMIM 613280), which is caused by mutations in SLC30A10, a Mn exporter required for biliary excretion of Mn (Tuschl et al., 2012a,b). Brain magnetic resonance imaging (MRI) appearances of patients with either disorder are indistinguishable for the hyperintensity of both the basal ganglia, particularly the globus pallidus, and the white matter on T1-weighted imaging (Tuschl et al., 2012b, 2016). Although patients with HMNDYT1 show significant improvement of neurological symptoms upon treatment initiation (Tuschl et al., 2008, 2012b), individuals with HMNDYT2 have variable treatment responses, with some patients experiencing a worsening of their movement disorder (Tuschl et al., 2016; Marti-Sanchez et al., 2018). The reasons for the difference in treatment response are poorly understood.

Although an essential trace metal, excess Mn acts as a neurotoxicant. Environmental Mn overexposure leads to preferential Mn accumulation in the globus pallidus, similar to that observed in inherited Mn transporter defects, and causes manganism, a Parkinsonian movement disorder characterised by bradykinesia, akinetic rigidity and dystonia, accompanied by psychiatric disturbances (Blanc, 2018; Chen et al., 2018). Despite the recognised role of Mn in neurodegenerative disease processes, the mechanisms related to Mn neurotoxicity remain poorly understood. The clinical similarities between manganism and Parkinson's disease (PD) suggest that dopaminergic signalling is impaired upon Mn toxicity. However, in manganism, dopaminergic neurons within the substantia nigra are intact and the response to L-3,4-dihydroxyphenylalanine (L-DOPA), the mainstay of treatment in PD, is poor (Koller et al., 2004). Glutamatergic excitotoxicity as well as altered gamma-aminobutyric acid (GABA) signalling have also been proposed to underlie Mn-associated neurodegeneration (Caito and Aschner, 2015). Indeed, Mn toxicity is likely mediated by a number of processes including oxidative stress, impaired mitochondrial function, protein misfolding and aggregation and neuroinflammation (Martinez-Finley et al., 2013; Tjalkens et al., 2017).

We have recently established and characterised a zebrafish loss-of-function mutant *slc39a14^U801/U801^* (herein referred to as *slc39a14^−/−^*) that closely resembles the human phenotype with systemic accumulation of Mn, particularly in the brain (Tuschl et al., 2016). Homozygous mutants develop increased susceptibility to Mn toxicity and impaired locomotor behaviour upon Mn exposure. Mn levels can be lowered through chelation with Na_2_CaEDTA similar to what is observed in human patients (Tuschl et al., 2016).

In this study, we performed RNA sequencing on individual *scl39a14^−/−^* larvae and their unaffected siblings to identify novel potential targets of Mn toxicity. Furthermore, we determined the transcriptional signature elicited in response to MnCl_2_ treatment in *scl39a14^−/−^* and their unaffected sibling larvae. Our results provide evidence that, in addition to Mn neurotoxicity, partial Mn deficiency that was corrected upon Mn treatment is a prominent feature of *slc39a14* loss-of-function. We determined that Ca^2+^ dyshomeostasis is a likely key event in both Mn deficiency and overload. Mn neurotoxicity is further associated with activation of the unfolded protein response (UPR), oxidative stress, mitochondrial dysfunction, apoptosis, autophagy and disruption of proteostasis. These changes accompany impaired neuronal activity within the telencephalon and optic tectum, as well as associated behaviours, of *slc39a14^−/−^* zebrafish.

## RESULTS

### Transcriptome analysis of *slc39a14^−/−^* mutants identifies increased sensitivity to Mn toxicity as well as Mn deficiency effects

**Fig. 1.**
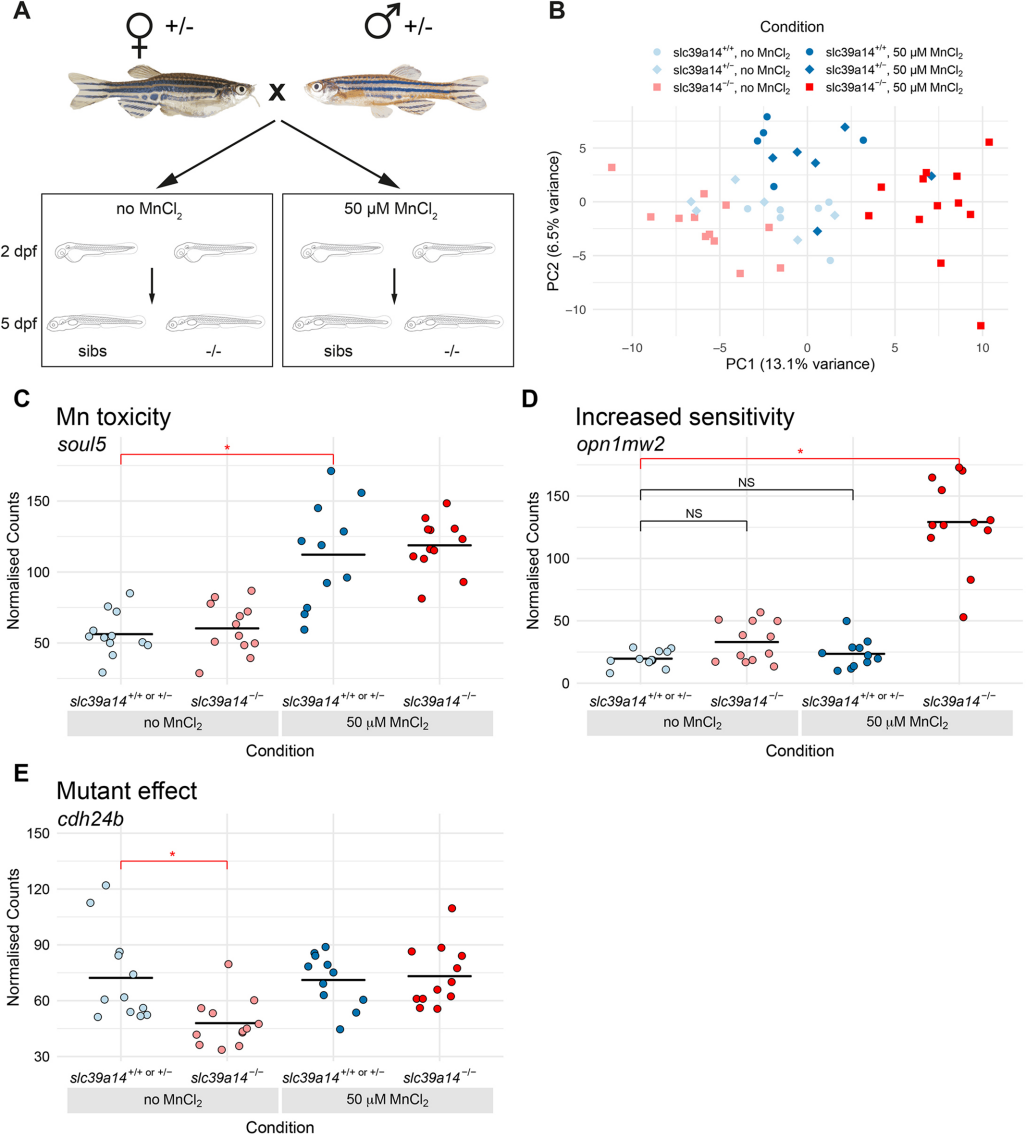
**DeTCT analysis identifies three groups of differentially expressed genes.** (A) Diagram of the experiment. Embryos from a *slc39a14^+/−^* incross were either left unexposed or exposed to 50 µM MnCl_2_ from 2 to 5 dpf. (B) Principal component analysis of the samples. Principal component (PC) 1 is plotted on the *x*-axis and PC2 on the *y*-axis. Samples belonging to the same condition are grouped together. Circles represent wild-type embryos, diamonds represent heterozygotes and squares represent homozygote mutants. Unexposed sibling embryos are indicated in light blue and MnCl_2_-exposed embryos are in dark blue. Unexposed mutants are coloured light red and exposed mutants are dark red. (C) Group 1 (Mn toxicity) genes were defined as those with a significant difference between exposed and unexposed siblings (red bar with asterisk). An example plot of normalised counts for the *soul5* gene is shown. The colour scheme for C-E is the same as in B. (D) Group 2 (Increased sensitivity) genes were defined as those with a significant difference between exposed mutants and unexposed siblings (red bar with asterisk), but without significant differences between either unexposed mutants or exposed siblings when compared to unexposed siblings (black bars labelled NS, not significant). An example plot of normalised counts for the *opn1mw2* gene is shown. (E) Group3 (Mutant effect) was defined as genes with a significant difference between unexposed mutants and unexposed siblings (red bar with asterisk). Example plot of normalised counts for the *cdh24b* gene. *P<0.05 determined using the Wald test (see Materials and Methods).

To investigate the transcriptional profiles of *slc39a14^−/−^* mutants in the absence and presence of Mn treatment, embryos from a heterozygous incross were split into two groups and either raised under standard conditions (subsequently referred to as unexposed), or treated with 50 µM MnCl_2_ from 2 to 5 days post fertilisation (dpf) (Fig. 1A). We have previously shown that this concentration elicits a pronounced locomotor phenotype in homozygous mutant larvae compared to their siblings (Tuschl et al., 2016). We then carried out transcriptional profiling of individual 5 dpf larvae using 3′ tag sequencing (differential expression transcript counting technique, DeTCT) (Collins et al., 2015). Principal component analysis (PCA) showed an effect of homozygosity and treatment status, but no difference between heterozygous and wild-type individuals (Fig. 1B, Table S1). We therefore pooled the wild-type and heterozygous embryos in the analysis for better statistical confidence and simplicity.

Analysis of differentially expressed genes between the four conditions produced three groups of genes, each with a characteristic expression profile. The first group consisted of genes that were differentially expressed in MnCl_2_-exposed siblings compared with their unexposed siblings, and represent a response to an increased concentration of Mn in the embryos (Fig. 1C, Mn toxicity). The second group consisted of genes that show increased sensitivity to Mn in *slc39a14^−/−^* mutants. These are defined as genes that are differentially expressed in MnCl_2_-exposed mutants compared with their unexposed siblings, but not differentially expressed in unexposed mutants compared with their unexposed siblings or exposed siblings compared with their unexposed siblings (Fig. 1D, Increased sensitivity). The third group was composed of genes that were differentially expressed in unexposed mutants compared with their unexposed siblings (Fig. 1E, Mutant effect). We then considered these three groups of genes in turn (see Table 1 for examples and Tables S2 and S3 for the top ten upregulated and downregulated genes and the highest *P*-values from each differentially expressed gene list).

**Table 1.**
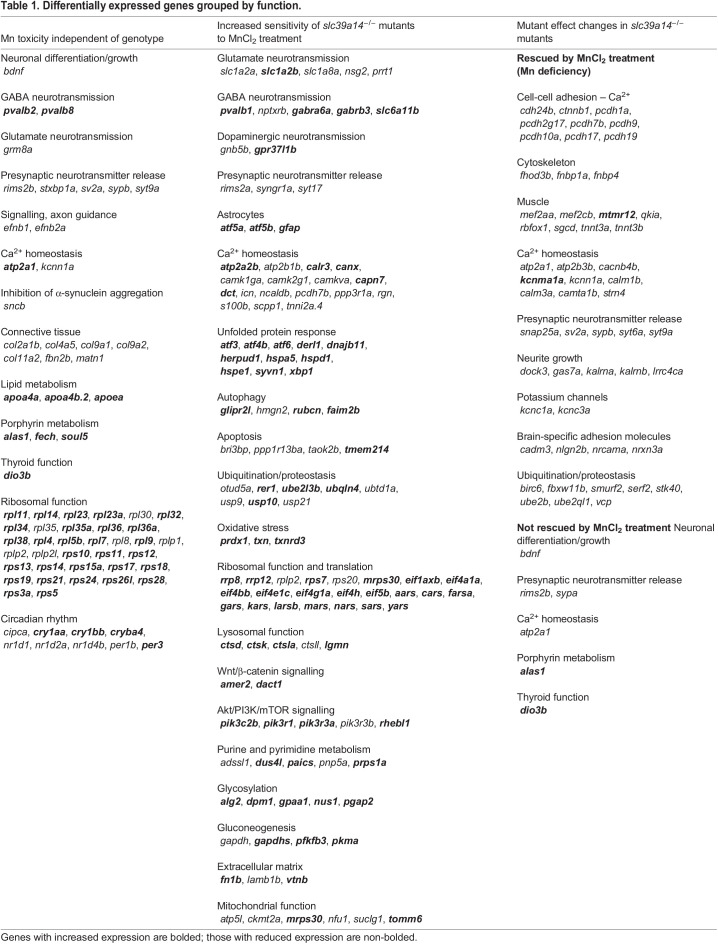
Differentially expressed genes grouped by function.

### Mn toxicity causes genotype-independent differential gene expression

**Fig. 2.**
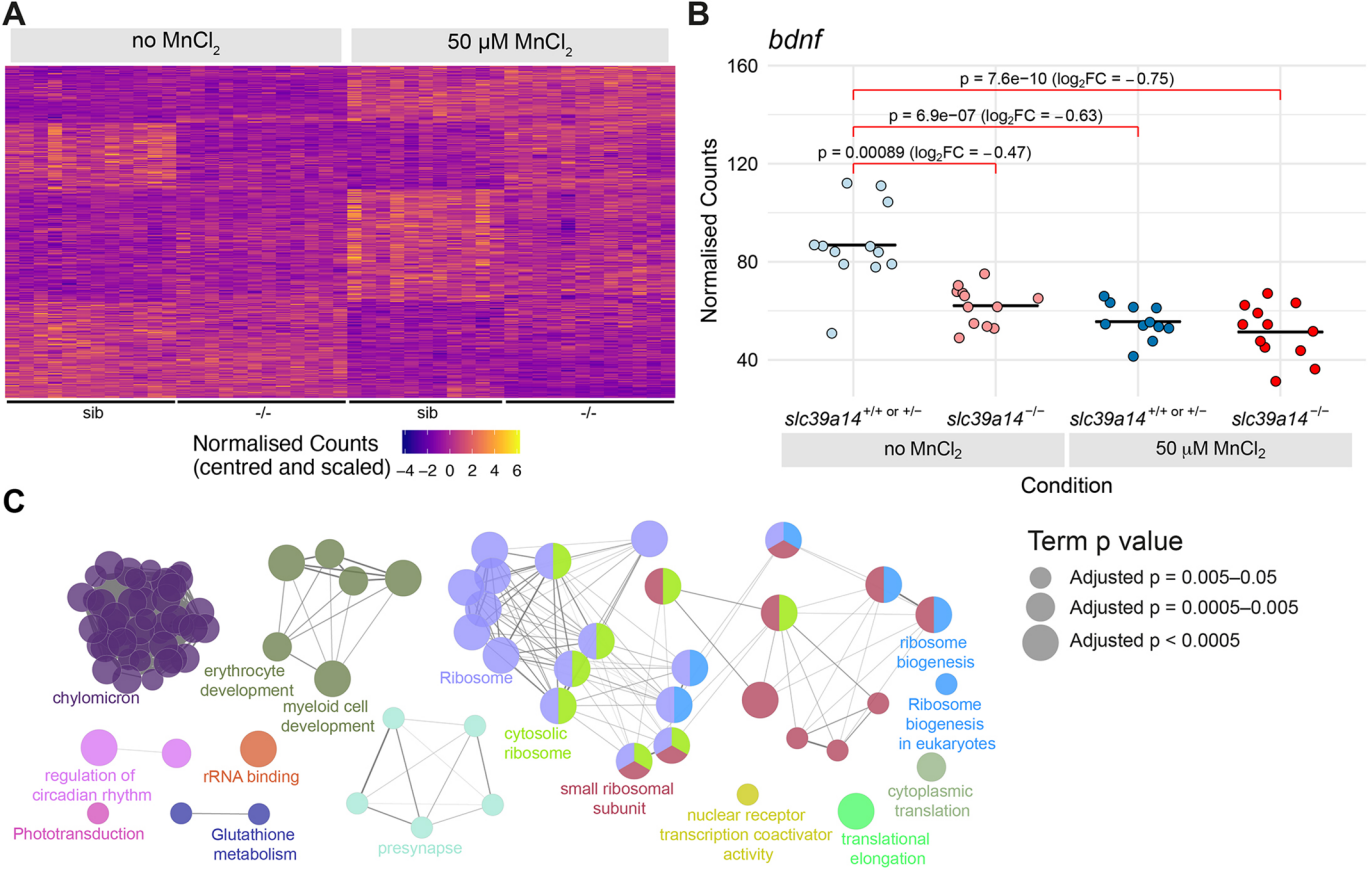
**Manganese overexposure causes neurotoxicity and metabolic defects in *slc39a14^+/+^ or slc39a14^+/−^* embryos.** (A) Heatmap of the expression of all 328 genes with a significant difference between exposed and unexposed siblings (Group 1 – Mn toxicity, Table S1). Each row represents a different gene and each column is a sample. Mutant embryos are displayed for completeness; however, the group of genes is defined by the response in siblings only. The normalised counts for each gene have been mean centred and scaled by dividing by the standard deviation. (B) Plot of the normalised counts for each sample of the gene *bdnf* in Group 1. Unexposed sibling embryos are indicated in light blue and MnCl_2_-exposed ones are in dark blue. Unexposed mutants are coloured light red and exposed mutants are dark red. FC, fold change. Wald test test was used to determine significance. (C) Enrichment of Gene Ontology (GO) terms associated with the genes in A. Diagram produced using the CytoScape ClueGO app. Nodes represent enriched GO terms and edges connect GO terms that have annotated genes in common. Different components of the network are coloured according to the categories labelled on the diagram. The sizes of the circles represent the adjusted *P*-values for each GO term as indicated on the right (Wald test). See Fig. S1 for GO enrichment split by up- and downregulation.

MnCl_2_ treatment caused differential expression of 328 genes independent of the genotype (comparing MnCl_2_-exposed and unexposed siblings) (Fig. 2A, Table 1; Table S1). Among them is *brain-derived neurotrophic factor* (*bdnf*), a previously reported read-out for Mn exposure (Zou et al., 2014), that also showed diminished expression in untreated mutants compared to their siblings (Fig. 2B). BDNF signalling has been linked to the maturation of Parvalbumin-positive cells, mainly GABAergic interneurons (Fairless et al., 2019). However, Parvalbumin-encoding genes were more highly expressed upon Mn exposure in mutants (*pvalb1*, *pvalb2* and *pvalb8*) as well as their siblings (*pvalb2* and *pvalb8*).

Among other brain-expressed genes affected by MnCl_2_ exposure were those involved in synaptic vesicle function (*rims2b*, *stxbp1a*, *sv2a*, *sypb* and *syt9a*) and genes encoding the Metabotropic glutamate receptor 8a (*grm8a*), β-Synuclein (*sncb*) and Ephrin-B membrane proteins (*efnb1* and *efnb2a*), all of which had decreased expression (Table 1).

Analysis of annotations to Gene Ontology (GO) terms (Fig. 2C; Table S4; Fig. S1 for GO enrichment split by up- and downregulation) showed enrichment of terms related to lipid metabolism (driven by upregulation of, for example, *apoa4b.2*, *apoa4a* and *apoea*), blood cell development (upregulation of *alas1*, *fech* and *soul5*), translation (35 ribosomal protein-encoding genes, most of which were upregulated) and circadian rhythm (upregulation of *cry1aa*, *cry1bb*, *cryba4* and *per3*). These findings were similar to previous reports in which links between Mn toxicity and lipid metabolism (Luo et al., 2020), circadian clock gene regulation (Li et al., 2017), haem-enzyme biogenesis (Chino et al., 2018) and protein biosynthesis (Hernandez et al., 2019) have been described.

Mn is important for connective tissue integrity and bone mineralisation as a constituent of metalloenzymes and enzyme activator (Sirri et al., 2016; Zofkova et al., 2017). Consistent with its role in connective tissue maintenance, transcriptome analysis confirmed that Mn exposure in zebrafish led to reduced expression of multiple connective tissue-related genes (*col2a1b*, *col4a5*, *col9a1a*, *col9a2*, *col11a2*, *dcn*, *fbn2b* and *matn1*).

### *slc39a14^−/−^* mutants show increased sensitivity to MnCl_2_ treatment

**Fig. 3.**
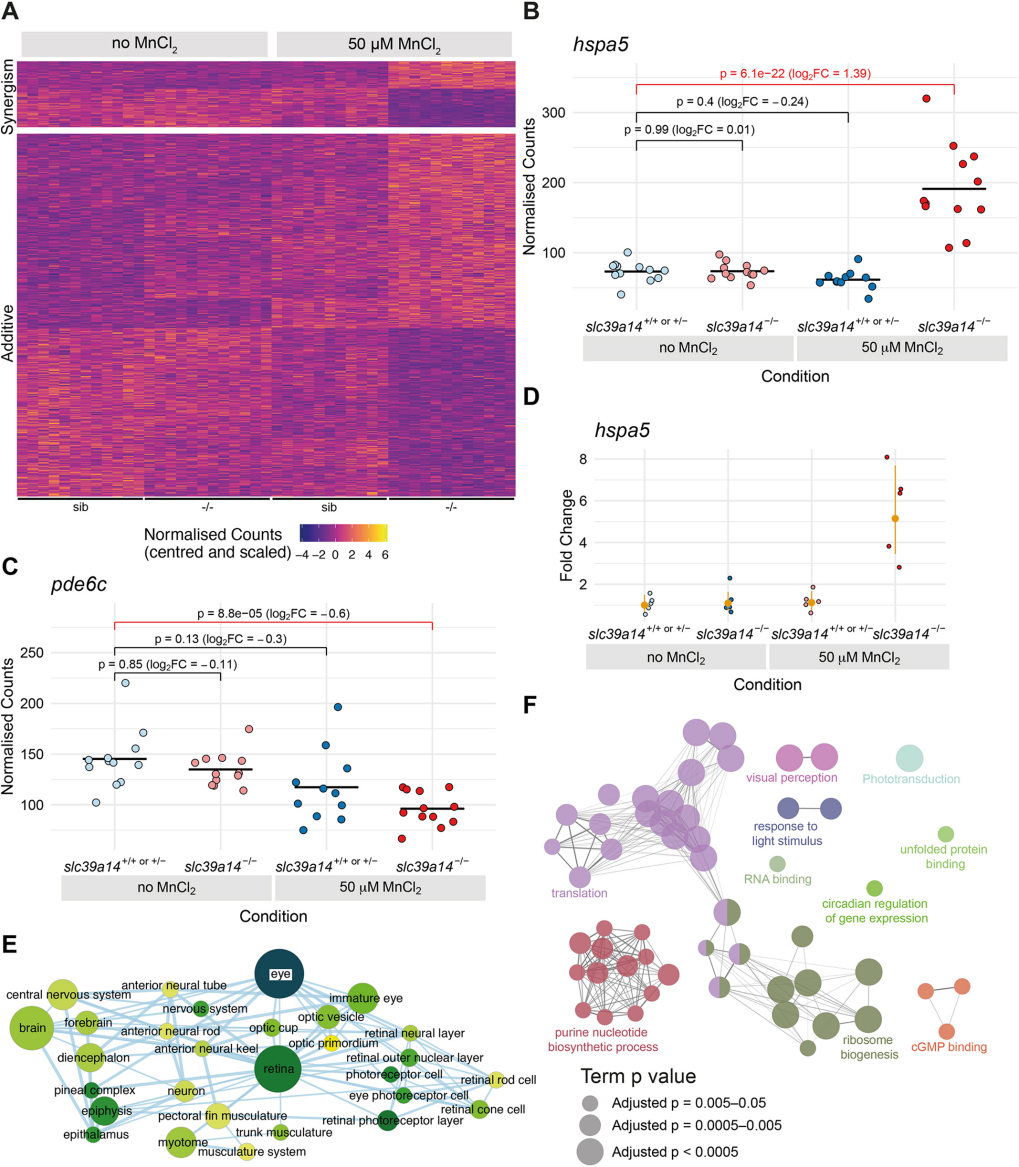
**Effect of Mn treatment in *slc39a14^−/−^* mutants.** (A) Heatmap of the expression of all 613 genes with a significant difference between exposed mutant and unexposed sibling embryos, but without without significant effects of treatment or genotype. The heatmaps are split into genes that show either synergistic or additive effects of the individual genotype and treatment effects. Each row represents a different gene and each column is a sample. The normalised counts for each gene have been mean centred and scaled by dividing by the standard deviation. (B) Example of a gene (*hspa5*) with a synergistic effect of treatment and genotype. The difference between the exposed mutants and unexposed siblings cannot be explained by adding together the separate effects of Mn treatment and the *slc39a14^−/−^* mutation. Unexposed sibling embryos are indicated in light blue and MnCl_2_-exposed embryos are in dark blue. Unexposed mutants are coloured light red and exposed mutants are dark red. (C) Example of a gene (*pde6c*) that has an additive effect of treatment and genotype. The difference between exposed mutants and unexposed siblings is consistent with adding together the two sub-threshold effects of treatment and genotype produce. Colour scheme is as in B. FC, fold change. Wald test was used to determine significance in B,C. (D) qRT-PCR showed comparable gene expression changes for *hspa5* as for the single-embryo sequencing dataset. The individual samples are displayed as fold change relative to the mean value for unexposed siblings, and the mean and 95% confidence intervals for each condition indicated are in orange. Compare with B. (E) Enrichment Map network of the Zebrafish Anatomy Ontology (ZFA) enrichment results. Each node represents an enriched ZFA term and the edges join nodes that have overlapping genes annotated to them. The width of each edge is proportional to the amount of overlap; nodes are coloured by −log_10_[adjusted *P*-value] (Wald test); and the size represents the number of significant genes annotated to the term. (F) ClueGO network diagram of the enrichment of GO terms. Nodes represent enriched GO terms and edges connect nodes that share annotations to the significant genes. Different components of the network are coloured according to the categories as labelled on the diagram. The sizes of the circles represent the adjusted *P*-values for each GO term as indicated below (Wald test).

Our analysis showed that 613 genes were differentially expressed in MnCl_2_-exposed mutants compared with their unexposed siblings, with no significant expression changes in either unexposed mutants or their exposed siblings. Therefore, these were genes that showed increased sensitivity to MnCl_2_ exposure in *slc39a14^−/−^* mutant larvae (Fig. 3A, Table 1). Of these genes, 15% (95/613) also had a significant genotype-treatment interaction effect, meaning that there was a synergistic effect on gene expression in mutant embryos with MnCl_2_ treatment – that is, the combined estimated effects of genotype and MnCl_2_ treatment alone were significantly less than the estimated log2 fold change for MnCl_2_-exposed mutants when compared with their unexposed siblings (Fig. 3B; see Table S1 for synergistic genes in bold). The remaining genes (518/613) showed expression changes consistent with additive effects of the sub-significance threshold responses to genotype and MnCl_2_ exposure alone (Fig. 3C). Results from the transcriptome analysis were validated by quantitative reverse transcription PCR (qRT-PCR) for a subset of six genes (*bdnf*, *gnat2*, *hspa5*, *opn1mw2*, *pde6ha* and *prph2b*) using RNA extracted from equivalent embryos in a different experiment (Fig. 3D,E; Fig. S2, Table S5). Changes in gene expression observed by qRT-PCR for all six genes were consistent with the results obtained from transcript counting (compare Fig. 3B with Fig. 3D and Fig. 3C with Fig. 3E).

Enrichment of zebrafish anatomy (ZFA) terms showed that genes differentially expressed upon MnCl_2_ exposure in *slc39a14^−/−^* mutants were disproportionately expressed in the nervous system, including the eye (Fig. 3F; Fig. S3, Table S6). This was confirmed by the enrichment of GO terms such as visual perception and phototransduction, associated with genes that were downregulated (Fig. 3F; Fig. S1). Also enriched were terms related to the ribosome, translation and the UPR, suggesting effects on protein synthesis and folding (Fig. 3F; Fig. S1, Table S4).

### Increased sensitivity of *slc39a14^−/−^* mutants to MnCl_2_ treatment leads to Mn neurotoxicity

Enriched ZFA terms identified in MnCl_2_-exposed *slc39a14^−/−^* mutants that were not present in siblings showed a high number of differentially expressed genes in the nervous system (Table S6), confirming the role of increased Mn levels in neurotoxicity. We found *slc1a2a*, encoding the astrocytic glutamate transporter excitatory amino acid transporter (EAAT2), to be the fifth most highly and significantly downregulated gene upon MnCl_2_ exposure (Tables S2 and S3). A role for astrocyte-mediated Mn neurotoxicity and neuroinflammation was further suggested by increased expression of the astrocyte-related genes *atf5a*, *atf5b* and *gfap*. In addition, expression of the teleost-specific glutamate transporters *slc1a2b* (upregulated) and *slc1a8a* (downregulated) was altered, pointing towards involvement of the glutamate-glutamine cycle in Mn neurotoxicity. Two genes required for the regulation of ionotropic AMPA-type glutamate receptors (*nsg2* and *prrt1*) also showed diminished expression in MnCl_2_-treated mutants (Table 1).

Furthermore, we observed increased expression of *slc6a11b*, encoding a GABA uptake transporter, as well as the Parvalbumin-encoding gene (*pvalb1*) present in GABAergic interneurons. Expression of the GABA-A receptor-encoding genes *gabra6a* and *gabrb3* and the Neuronal pentraxin receptor-encoding gene *nptxrb*, which is expressed in Parvalbumin-positive interneurons (Kikuchihara et al., 2015), was reduced.

Despite the assumption that abnormal dopamine signalling is a major player in Mn neurotoxicity (Guilarte and Gonzales, 2015), only two genes linked to dopamine, *gnb5b* (downregulated) and *gpr37l1b* (upregulated), both of which encode proteins that interact with neurotransmission via the Dopamine D2 receptor (Octeau et al., 2014; Hertz et al., 2019), were differentially expressed.

In order to assess the effects of Mn neurotoxicity on neuronal function, we performed *in situ* hybridisation chain reaction to map *gad1b* mRNA in MnCl_2_-exposed wild-type and mutant siblings. *gad1b* was chosen because Mn preferentially accumulates in the globus pallidus, a region that is particularly rich in GABAergic projections, both in individuals with Mn overexposure and those with inherited Mn transporter defects. However, spatial *gad1b* expression analysis did not suggest changes in GABAergic signalling and brain structure (Fig. S4).

### MnCl_2_ exposure alters resting-state neuronal activity and locomotor behaviour

**Fig. 4.**
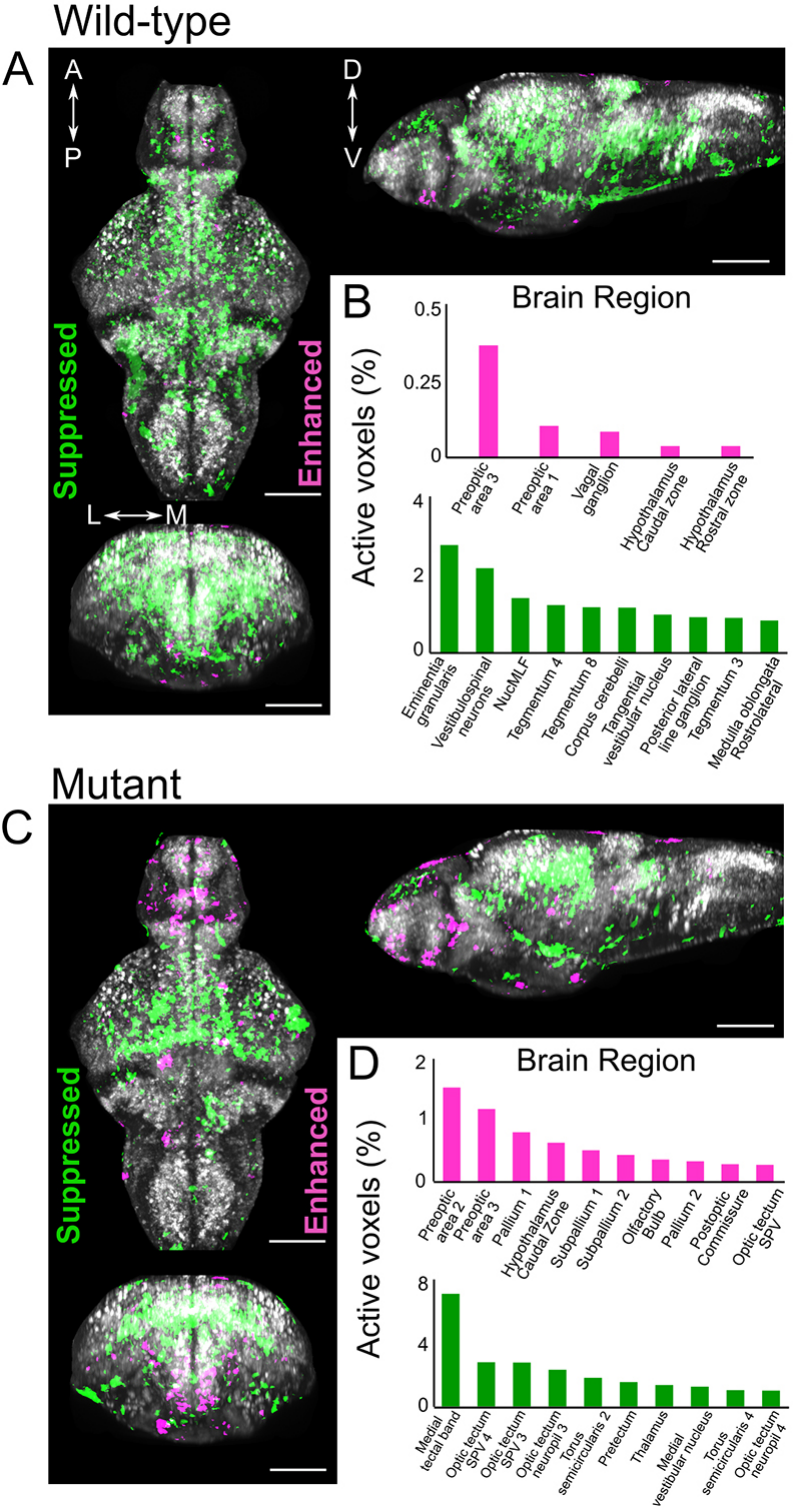
**MnCl_2_ treatment alters neuronal activity in both wild-type and *slc39a14^−/−^* larvae.** (A-D) *Z*-projection of *cfos* mRNA expression in the brain of wild-type (A) and homozygous mutant larvae (C) at 6 dpf following treatment with 50 µM MnCl_2_ from 2 dpf. B and D list the brain regions with enhanced (magenta) and suppressed (green) neuronal activity by genotype. A, anterior; P, posterior; D, dorsal; V, ventral; L, lateral; M, medial. Scale bars: 100 µm.

*cfos* (officially known as *fosab*) is an immediate-early gene induced in response to neuronal activity, and thus, we performed *in situ* hybridisation chain reaction to map changes in *cfos* expression in response to MnCl_2_ exposure as a proxy for identifying resting-state changes in neuronal activity. We observed pronounced alterations in *cfos* expression in both MnCl_2_-treated wild-type and mutant siblings (Fig. 4A-D). Consistent with the increased sensitivity to Mn neurotoxicity suggested by RNA sequencing, homozygous mutant larvae showed more extensive changes in *cfos* expression compared to their siblings. Specifically, enhanced expression of *cfos*, reflecting increased neuronal activity, was particularly evident within the telencephalon in mutant versus wild-type larvae, whereas lower expression was observed within the optic tectum of mutants (Fig. 4D).

**Fig. 5.**
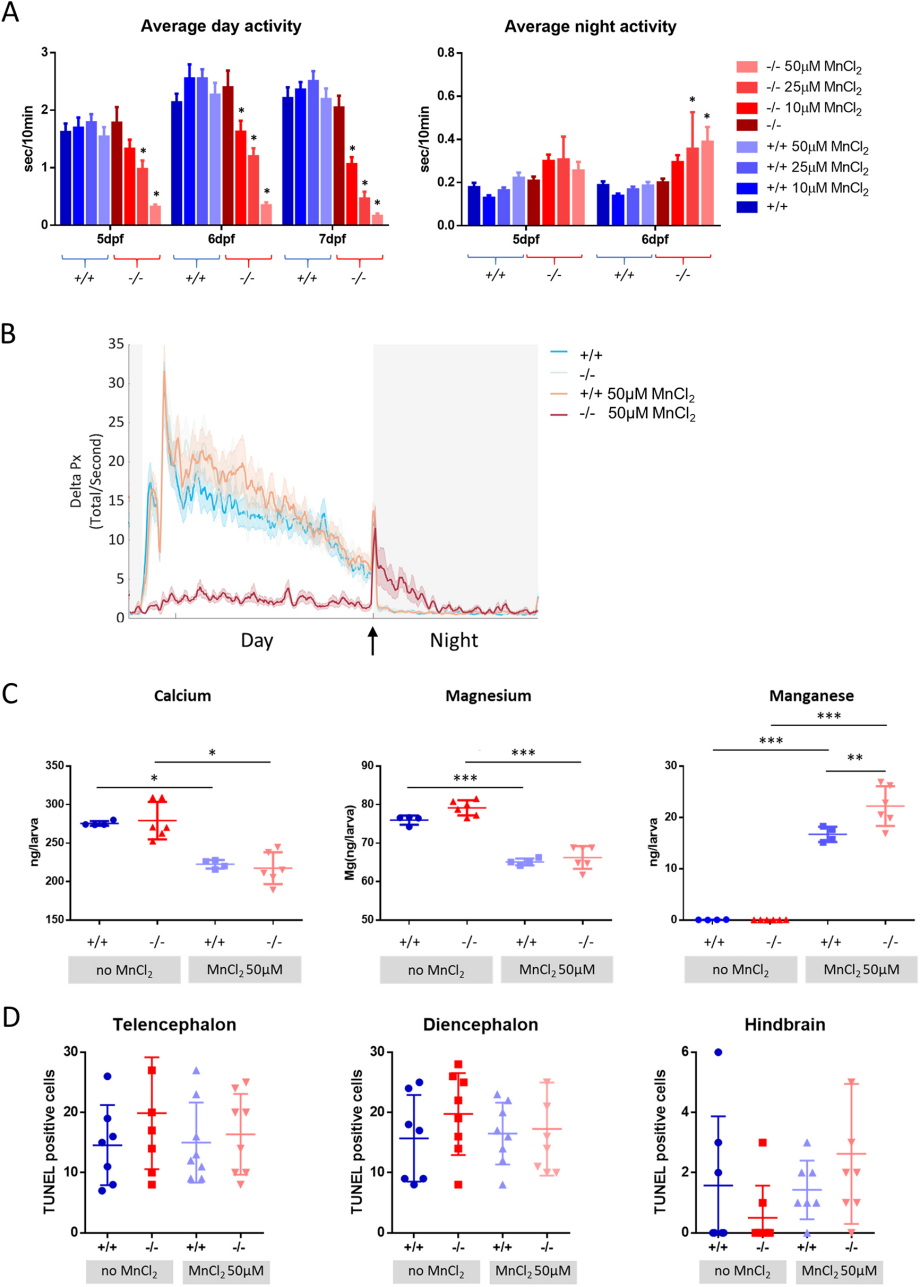
**MnCl_2_ treatment causes locomotor abnormalities and** Ca^2+^ **dyshomeostasis.** (A) Average locomotor activity of wild-type (blue) and *slc39a14^−/−^* larvae (red) during the day and night in response to increasing concentrations of MnCl_2_. Data are presented as mean±s.e.m. (two-way ANOVA with Tukey's posthoc test; *P<0.05), *n*=24 larvae per group. The *y*-axes represent the average activity of larval zebrafish in seconds per 10 minutes. (B) Frame-by-frame analysis of the locomotor activity of wild-type and *slc39a14^−/−^* larvae at 6 dpf that were unexposed and exposed to 50 µM MnCl_2_. White shading representing the day (lights ON) and grey shading represents the night (lights OFF). The arrow indicates the ‘lights OFF’ switch. Summed and smoothed Δ pixels traces (Delta Px) are shown as mean±s.e.m. (bold lines and shaded surrounding areas). *n*=24 larvae per group. (C) Calcium, magnesium and manganese concentrations determined by ICP-MS in untreated and MnCl_2_ (50 µM)-treated wild-type and *slc39a14^−/−^* larvae. Data are presented as mean±s.d. (one-way ANOVA with Tukey's post hoc test; *P<0.05; **P<0.01; ***P<0.001). (D) Apoptotic cell death upon MnCl_2_ exposure in both wild-type (blue) and mutant (red) larvae at 5 dpf detected by TUNEL staining in the telencephalon, diencephalon and hindbrain. Data are presented as mean±s.d.

We next tracked the locomotor behaviour of unexposed and MnCl_2_-exposed wild-type and mutant larvae from 4 to 7 dpf on a 14:10 h light-dark cycle. Homozygous mutants showed a dose-dependent reduction in average locomotor activity during the day and increased locomotor activity during the night, whereas wild-type larvae remained unaffected by MnCl_2_ exposure (Fig. 5A; Table S7). Wild-type fish sharply increased their locomotor activity immediately following lights OFF and gradually, over several minutes, returned to baseline locomotor activity, a behaviour known as the visual motor response (VMR) (Burton et al., 2017). Frame-by-frame analysis of larval locomotion showed that *slc39a14^−/−^* zebrafish had a preserved VMR but showed hyperlocomotion throughout the first few hours following lights OFF, with larvae returning to baseline activity only towards the second half of the night (Fig. 5B).

### Increased sensitivity of *slc39a14^−/−^* mutants to MnCl_2_ treatment is associated with gene expression changes affecting Ca^2+^ and protein homeostasis, and the UPR

Mn toxicity is known to cause protein misfolding and aggregation (Angeli et al., 2014; Harischandra et al., 2019b) and, as previously shown for Mn overexposure in *Caenorhabditis elegans* (Angeli et al., 2014), multiple genes involved in the UPR had increased expression in *slc39a14^−/−^* mutants, with *hspa5*, *atf3* and *xbp1* observed to be the most highly and significantly upregulated genes upon MnCl_2_ treatment (Table 1; Tables S2 and S3). This is supported by transcription factor motif-enrichment analysis using Hypergeometric Optimization of Motif EnRichment analysis (HOMER) (Heinz et al., 2010), which showed that the dysregulated genes are enriched for Chop/Atf4-binding sites among others (Fig. S3, Table S8). Degradation of misfolded and aggregated proteins occurs via the ubiquitin-proteasome system within the cytosol (Tamas et al., 2014), and MnCl_2_-exposed *slc39a14^−/−^* mutants showed gene expression changes linked to ubiquitination (Table 1). Ca^2+^ homeostasis within the endoplasmic reticulum (ER) plays a major role during the UPR, and vice versa (Groenendyk et al., 2021). Potentially linked to the UPR, over a dozen Ca^2+^-associated/dependent genes were differentially expressed in MnCl_2_-treated *slc39a14^−/−^* mutants (Table 1; Table S1).

Given the observed changes in the expression of Ca^2+^-linked genes, we next assessed total Ca, Mg and Mn levels in both wild-type and mutant larvae by inductively coupled plasma mass spectrometry (ICP-MS). Consistent with a disturbance in Ca^2+^ homeostasis, we found that MnCl_2_ treatment in both wild-type and mutant larvae led to a marked decrease in total Ca and Mg levels (Fig. 5C). As previously observed, Mn accumulation was much greater in mutant larvae (Fig. 5C). These results confirm that Mn overload leads to Ca^2+^ dyshomeostasis that is associated with changes in expression of key genes responsible for Ca^2+^ regulation.

Activation of the UPR as well as Ca^2+^ dyshomeostasis can promote apoptosis and autophagy. Concordantly, genes involved in autophagy and apoptosis were differentially expressed (Table 1). In particular, *faim2b*, which encodes the recently identified regulator of autophagy FAIM2B (Hong et al., 2020), was the third most highly upregulated gene in MnCl_2_-exposed *slc39a14^−/−^* mutants (Table S2). Also, the expression of *rubcn*, encoding a beclin 1 interactor and responsible for autophagy initiation (Liu et al., 2019), was increased upon MnCl_2_ exposure.

To further explore whether increased apoptotic cell death might be responsible for the high number of downregulated genes observed upon MnCl_2_ exposure, we performed terminal deoxynucleotidyl transferase dUTP nick end labelling (TUNEL) staining on brains from unexposed and MnCl_2_-exposed wild-type and mutant larvae (Fig. 5D). However, there was no difference in the number of TUNEL-positive cells between unexposed and MnCl_2_-exposed larvae of either genotype, suggesting that functional rather than neurodegenerative changes are responsible for Mn neurotoxicity effects.

Oxidative stress and mitochondrial dysfunction are prominent features of Mn toxicity (Smith et al., 2017; Harischandra et al., 2019a). Consistent with this observation, essential genes of the thioredoxin/peroxiredoxin system (*prdx1*, *txn* and *txnrd3*) were activated in MnCl_2_-exposed *slc39a14^−/−^* mutants. Likewise, genes related to mitochondrial function showed differential expression in MnCl_2_-treated mutants (Table 1). Our data therefore further support a role of mitochondrial impairment in Mn-induced neurotoxicity.

### Increased sensitivity of *slc39a14^−/−^* mutants to MnCl_2_ causes visual impairment

**Fig. 6.**
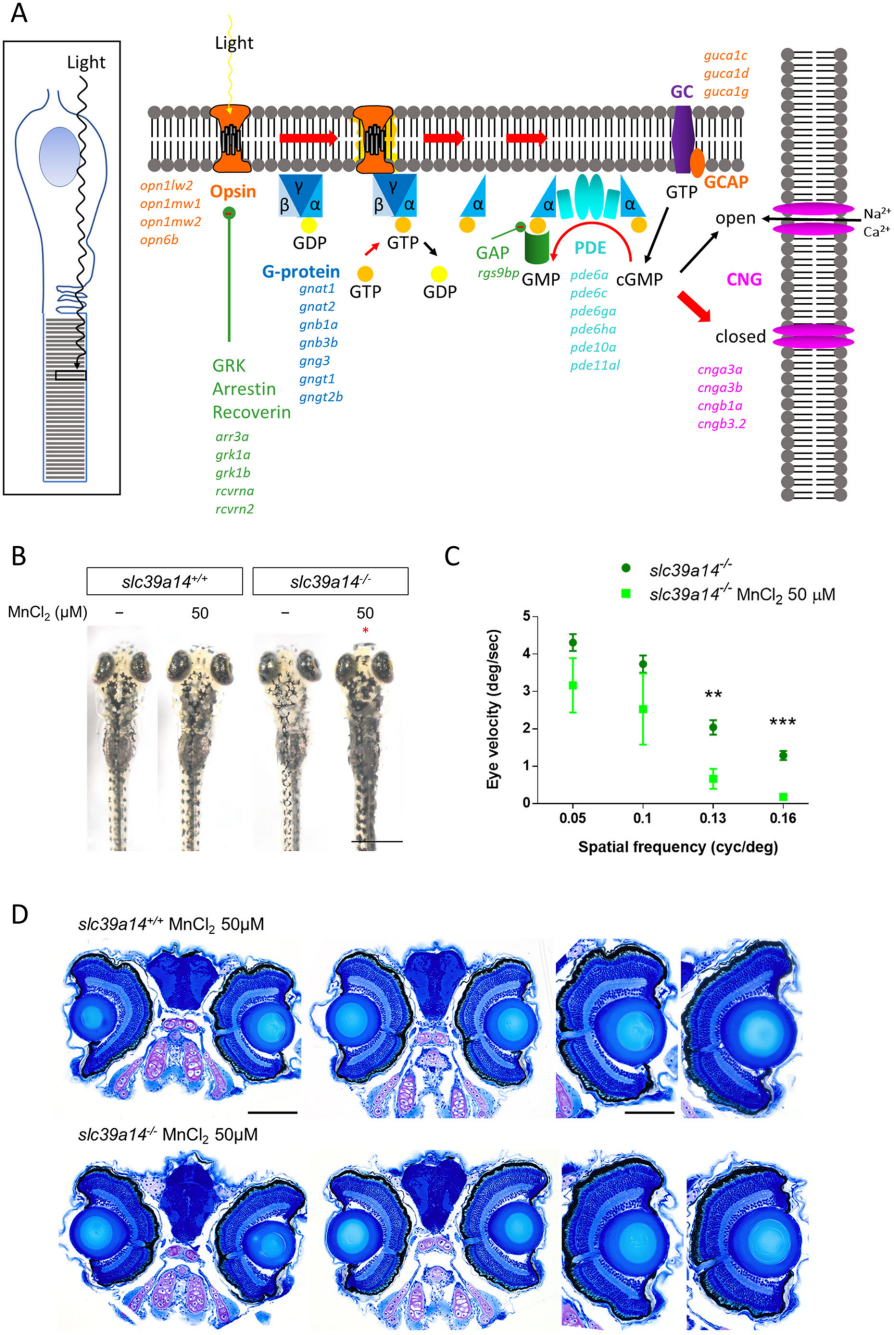
***slc39a14^−/−^* mutants develop a visual phenotype upon MnCl_2_ exposure.** (A) Schematic showing the process of phototransduction (Kaupp and Seifert, 2002) with the differentially expressed genes observed in MnCl_2_-exposed *slc39a14^−/−^* mutants indicated in italics. cGMP, cyclic guanosine monophosphate; CNG, cyclic nucleotide-gated non-selective cation channels; GC, guanylyl cyclase; GCAP, guanylate cyclase-activating protein; PDE, phosphodiesterase; GRK, G protein-coupled receptor kinase; GAP, GTPase-activating protein. (B) Dorsal views during light exposure of wild-type siblings (*slc39a14^+/+^*, left) and *slc39a14^−/−^* larvae (right) at 5 dpf that were unexposed and exposed to 50 µM MnCl_2_. Note the darker pigmentation of mutants exposed to MnCl_2_ (red asterisk). Scale bar: 500 µm. (C) Graph showing the eye velocity in response to moving stimuli of different spatial frequencies (average of both eyes) of *slc39a14^−/−^* larvae that were unexposed (dark green circles) and exposed to 50 µM MnCl_2_ (light green squares). Data are presented as mean±s.e.m. from five independent experiments (two-tailed unpaired Student's *t*-test; **P<0.01; ***P<0.001). (D) Histologic analysis of retinal sections with Richardson–Romeis staining of wild-type siblings (*slc39a14^+/+^*, top row) and *slc39a14^−/−^* larvae (bottom row) at 5 dpf exposed to 50 µM MnCl_2_. Scale bars: 200 µm (image of both eyes), 100 µm (image of single eye).

Consistent with the reduced *cfos* expression/neuronal activity observed within the optic tectum (Fig. 4), 30 genes involved in phototransduction were differentially expressed (27/30 genes were reduced) in MnCl_2_-exposed mutants but not in their siblings (Fig. 6A; Table S1). These included some of the most significantly upregulated genes such as *pde6ha*, *opn1mw2*, *opn1mw1* and *rcvrna* in the increased sensitivity group (Tables S2 and S3). Hence, we further examined the vision of *slc39a14^−/−^* mutants. In zebrafish, visual background adaptation (VBA), which is the ability to aggregate and disperse melanosomes in order to adapt their body pigmentation to the environment, requires retinal input and is impaired in blind larvae (Mueller and Neuhauss, 2014).

We observed that MnCl_2_-exposed *slc39a14^−/−^* mutant larvae lacked melanosome aggregation and remained dark following light exposure from 4 dpf, whereas exposed wild-type larvae and unexposed mutants demonstrated a normal VBA (Fig. 6B). Next, we analysed the optokinetic response (OKR) in homozygous *slc39a14^−/−^* larvae at 5 dpf after MnCl_2_ exposure. Exposed mutant larvae demonstrated a significant reduction in slow-phase eye velocity at high spatial frequencies, suggesting impaired visual acuity (Fig. 6C; Table S9). Retinal histology of mutant and MnCl_2_-exposed animals appeared normal, suggesting functional rather than overt structural deficits (Fig. 6D). In conclusion, the reduced expression of phototransduction genes in combination with reduced *cfos* expression/neuronal activity within the optic tectum, impaired VBA and OKR, as well as abnormal VMR reveal that Mn exposure in *slc39a14^−/−^* larvae leads to visual impairment.

### Most differentially expressed genes in unexposed *slc39a14^−/−^* mutants are rescued by Mn treatment suggesting Mn deficiency

**Fig. 7.**
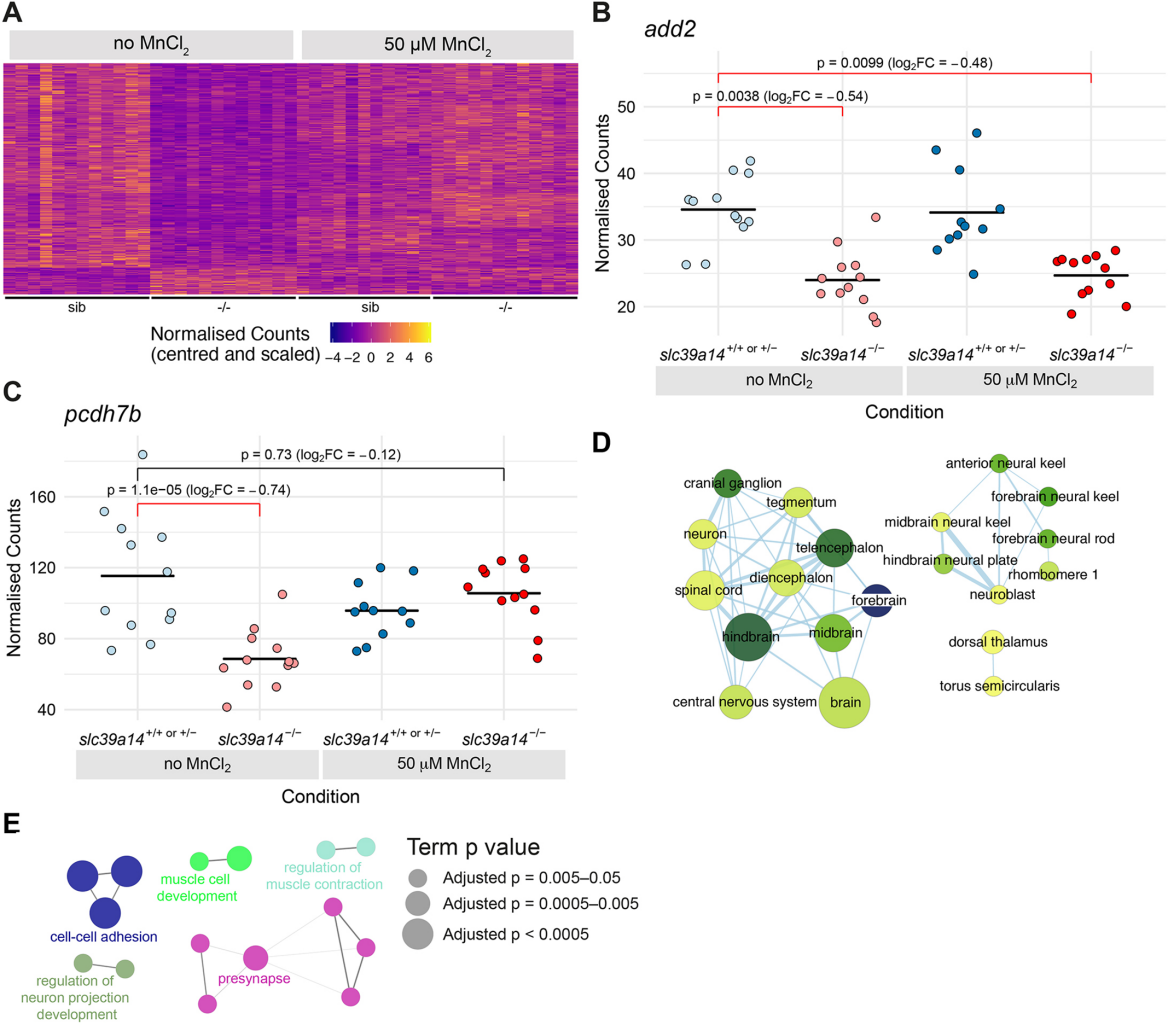
**Exogenous Mn restores normal expression of genes that are differentially expressed in unexposed *slc39a14^−/−^* mutants.** (A) Heatmap of the expression of 266 genes with a significant difference between unexposed mutants and unexposed siblings. Each row represents a different gene and each column is a sample. The normalised counts for each gene have been mean centred and scaled by dividing by the standard deviation. (B) Plot of normalised counts for the *add2* gene. Gene expression is decreased in both unexposed and MnCl_2_-exposed mutant embryos. Unexposed sibling embryos are indicated in light blue and Mn-exposed embryos are in dark blue. Unexposed mutants are coloured light red and exposed mutants are dark red. (C) Plot of normalised counts for the *pcdh7b* gene. There were decreased counts in the unexposed mutant embryos that were rescued back to wild-type levels upon 50 µM MnCl_2_ treatment. Colour scheme is as in B. FC, fold change. Wald test was used to determine significance in B,C. (D) Enrichment Map diagram of the enrichment of ZFA terms for the genes differentially expressed in unexposed mutants that are rescued by Mn treatment. Nodes represent enriched ZFA terms and edges connect nodes that share annotations to the significant genes. The width of each edge is proportional to amount of overlap, nodes are coloured by −log_10_[adjusted *P*-value] (Wald test) and the size represents the number of significant genes annotated to the term. (E) ClueGO network diagram of the enrichment of GO terms associated with the genes that are rescued by Mn treatment. Nodes represent enriched GO terms and edges connect nodes that share annotations to the significant genes. Different components of the network are coloured according to the categories as labelled on the diagram. The sizes of the circles represent the adjusted *P*-values for each GO term as indicated on the right (Wald test).

When compared to unexposed siblings, 266 genes showed significantly different expression due to the homozygous state alone (unexposed homozygous mutants versus unexposed unaffected siblings) (Fig. 7A; Table S1). Expression of 12% of these genes (31/266) was also significantly different between MnCl_2_-exposed mutants and their unexposed siblings (Fig. 7B). Seven of these genes overlapped with those differentially expressed in siblings upon MnCl_2_ exposure, suggesting that these genes were the most sensitive targets of Mn toxicity (*alas1*, *atp2a1*, *bdnf*, *crim1*, *dio3b*, *dip2ca* and *rims2b*). However, the majority (88%, 235/266) of differentially expressed genes in unexposed mutants were not significantly differentially expressed when comparing MnCl_2_-exposed mutants and their unexposed siblings (Fig. 7C). This suggests that the homozygous *U801* mutation creates an Mn deficiency leading to gene expression changes that return to levels observed in unexposed and unaffected siblings upon MnCl_2_ treatment.

Analysis of ZFA terms within this rescued set of genes demonstrated enrichment of terms related to the nervous system (Fig. 7D; Fig. S3, Table S6). For instance, brain-expressed genes that showed reduced expression upon Mn deficiency include those essential for synaptic function and vesicle formation (*snap25a*, *sv2a*, *sypb*, *syt6a* and *syt9a*), neurite and axonal growth (*dock3*, *gas7a*, *kalrna*, *kalrnb* and *lrrc4ca*) and potassium channels (*kcnc1a* and *kcnc3a*). GO term analysis linked differential gene expression to cell-cell adhesion and cell-cell interactions (Fig. 7E; Fig. S1, Table S4). Expression of seven protocadherin-encoding genes was altered with *pcdh7b* being the third most highly downregulated gene within this group. Protocadherins are Ca^2+^-dependent cell-adhesion proteins that are primarily expressed in the brain, where they regulate synapse maturation, function and plasticity (Mancini et al., 2020). In addition, the expression of several other Ca^2+^-associated genes returned to normal levels by Mn treatment, which were distinct from those changed due to Mn toxicity. These included genes encoding Ca^2+^ ATPases (*atp2a1* and *atp2b3b*), Ca^2+^ channels (*cacnb4b*), Ca^2+^-activated potassium channels (*kcnma1a* and *kcnn1a*), calmodulins (*calm1b* and *calm3a*) and calmodulin-binding proteins (*camta1b* and *strn4*). These results suggest that in addition to causing a systemic increase in Mn levels, the loss of *slc39a14* function might also result in local Mn deficiency with gene expression changes that can be rescued with exogenous Mn. Differentially expressed genes in both the Mn sensitivity and Mn rescue group link to Ca^2+^ regulation, suggesting that disturbed Mn homeostasis has significant consequences on Ca^2+^-dependent genes with a distinctly affected gene set for each group (Table 1).

## DISCUSSION

Transcriptional profiling of *slc39a14* mutant zebrafish identified distinct gene groups that are differentially expressed in physiological conditions and upon MnCl_2_ exposure. Consistent with the neurodegenerative phenotype observed in HMNDYT2 patients and the previously described accumulation of Mn in the brain of *slc39a14^−/−^* zebrafish mutants (Tuschl et al., 2016), the majority of differentially expressed genes map to the central nervous system (CNS) and the eye. Mn treatment leads to gene expression changes in both *slc39a14^−/−^* mutant and sibling zebrafish. However, the expression of a much greater number of genes changes in mutant larvae upon MnCl_2_ treatment than in treated non-mutant siblings, confirming an increased sensitivity to Mn toxicity that is consistent with previous observations (Tuschl et al., 2016). This is corroborated by the changes in brain activity, locomotor and visual behaviour observed in mutant larvae. Intriguingly, 88% (235/266) of differentially expressed genes in unexposed *slc39a14^−/−^* mutants normalised upon MnCl_2_ treatment. This suggests that Mn treatment in *slc39a14^−/−^* mutants rescues some of the transcriptomic changes observed in unexposed mutants, and implies that SLC39A14 loss leads to Mn deficiency, in parallel to the observed Mn accumulation.

### Unexposed *slc39a14^−/−^* mutants as well as MnCl_2_-treated mutants and siblings show evidence of Mn neurotoxicity

The mechanisms underlying Mn neurotoxicity are heterogenous, suggesting extensive roles for Mn in brain pathobiology (Soares et al., 2020). The neuronal subtypes affected by Mn neurotoxicity remain the subject of debate. In agreement with previous reports, we observed altered expression of genes involved in glutamatergic and GABAergic neurotransmission in MnCl_2_-treated *slc39a14^−/−^* mutants (Marreilha Dos Santos et al., 2017). The most highly and significantly downregulated genes included *slc1a2a*, encoding the astrocytic glutamate reuptake transporter EAAT2. Transcriptional repression of *SLC1A2* with subsequent impaired glutamate uptake and excitotoxicity has been observed in MnCl_2_-exposed human astrocytes (Rizor et al., 2021), suggesting that Mn neurotoxicity affects the glutamate-glutamine cycle.

In humans, Mn preferentially accumulates in the globus pallidus, a region that is particularly rich in GABAergic neurons (Sidoryk-Wegrzynowicz and Aschner, 2013; Tuschl et al., 2016). In MnCl_2_-treated *slc39a14^−/−^* zebrafish, the expression of genes encoding the GABA-A receptor (*gabra6a* and *gabrb3*) and the GABA reuptake transporter (*slc6a11b*) was reduced, similar to studies in rats where Mn exposure led to diminished GABA-A receptor mRNA expression and interfered with GABA uptake in astrocytes (Fordahl and Erikson, 2014; Ou et al., 2017). Increased expression of genes encoding Parvalbumin (*pvalb1*, *pbalb2* and *pvalb8*) in *slc39a14^−/−^* mutants and their siblings upon MnCl_2_ treatment might further indicate that GABAergic interneurons are a target of Mn neurotoxicity (Kikuchihara et al., 2015). Parvalbumin, a Ca^2+^-binding protein, can also bind Mn^2+^ with high affinity (Nara et al., 1994). Mn might therefore interact with Parvalbumin directly or via changes in Ca^2+^ homeostasis, which are clearly evident in *slc39a14^−/−^* zebrafish. Despite the observed gene expression changes related to GABAergic neurotransmission, the spatial localisation of *gad1b* mRNA expression was unchanged in both MnCl_2_-exposed wild-type and *slc39a14^−/−^* larvae. However, the observed marked alterations in *cfos* expression suggested altered neuronal activity in *slc39a14^−/−^* compared with wild-type fish. Enhanced expression/activity was evident within preoptic, hypothalamic, pallidal and subpallidal regions.

Because manganism resembles Parkinson's disease to some extent (e.g. both cause an akinetic movement disorder, albeit with distinct clinical features), it has long been hypothesised that dopaminergic neurons are affected by Mn neurotoxicity (Ijomone et al., 2016). However, transcriptome analysis of *slc39a14^−/−^* mutants provides little evidence that Mn neurotoxicity causes primary gene expression changes related to dopaminergic signalling.

Consistent with predominant accumulation of Mn in astrocytes rather than neurons (Tjalkens et al., 2017; Gorojod et al., 2018; Popichak et al., 2018), Mn exposure in *slc39a14^−/−^* mutants leads to increased expression of the astrocyte-related genes *atf5a*, *atf5b* and *gfap*, as well as the astrocyte-expressed glutamate and GABA uptake transporter genes *slc1a2* and *slc6a11b*, respectively, corroborating a role for glia in Mn neurotoxicity.

Although we observed gene expression changes linked to apoptosis, TUNEL staining did not reveal increased apoptotic cell death upon MnCl_2_ exposure. This suggests that Mn neurotoxicity initially and primarily causes deficits in neuronal function rather than neurodegeneration, which is in keeping with clinical observations that the neuronal phenotype of affected individuals is to some extent reversible (Tuschl et al., 2016).

### Mn toxicity in *slc39a14^−/−^* mutants is associated with Ca^2+^ dyshomeostasis, activation of the UPR and oxidative stress

Our results clearly indicate that Mn imbalance interferes with Ca^2+^ homeostasis and causes changes in expression of Ca^2+^-associated genes coupled with altered total Ca^2+^ levels. It is understood that Mn^2+^ can replace Ca^2+^ in its biologically active sites (Kalbitzer et al., 1978; Song et al., 2017) and disrupt Ca^2+^ homeostasis at the mitochondria and the ER, thereby affecting intracellular Ca^2+^ concentrations (Quintanar et al., 2012). Mn overexposure has previously been shown to disrupt neurotransmitter release via interaction with the SNARE complex and subsequent activation of calpain, a Ca^2+^-/Mn^2+^-activated neutral protease (Wang et al., 2018). MnCl_2_ treatment in *slc39a14^−/−^* mutants indeed affects the expression of genes encoding parts of the presynaptic neurotransmitter release machinery, suggesting that Mn neurotoxicity might be mediated through impaired presynaptic exocytosis. Whether this is facilitated via direct interaction of Mn with neurotransmitter release or via Ca^2+^ dysregulation needs to be determined in future studies. Nevertheless, our results provide evidence that Ca^2+^ dysregulation is a key feature of Mn neurotoxicity. This has also been shown for other neurodegenerative disorders including Parkinson's, Alzheimer's and Huntington's diseases, in which Ca^2+^ dyshomeostasis occurs upstream of protein aggregation (Jadiya et al., 2021).

Ca^2+^ homeostasis is maintained by the ER, the key organelle for regulating proteostasis (Wang et al., 2012). Ca^2+^ dysregulation is closely linked to the UPR and ER stress that is evident in MnCl_2_-exposed *slc39a14^−/−^* mutants, with upregulation of multiple UPR-associated genes. HOMER analysis also confirms enrichment of the Chop/Atf4 motif in MnCl_2_-treated mutants. This is consistent with previous studies that show increased expression of ATF6 and HSPA5 as well as increased *Xbp1* mRNA splicing in Mn-exposed brain slices (Xu et al., 2013).

In addition to Ca^2+^ dyshomeostasis, oxidative stress and mitochondrial dysfunction are shared characteristics among neurodegenerative disorders and metal toxicity (Harischandra et al., 2019a). Mn accumulates in mitochondria, where it leads to the generation of reactive oxygen species (ROS) (Rizor et al., 2021). ROS production can further exacerbate protein misfolding (Nakamura et al., 2021). Oxidative stress is highlighted in MnCl_2_-exposed *slc39a14^−/−^* mutants by the upregulation of the thioredoxin/thioredoxin reductase and peroxiredoxin system, similar to previous results in rats (Taka et al., 2012). ROS also cause apoptosis and autophagy via lysosomal membrane permeabilisation and cathepsin release (Gorojod et al., 2017; Wang et al., 2017; Porte Alcon et al., 2018; Zhi et al., 2019; Tinkov et al., 2021). In accordance, we observed changes in autophagy and cathepsin gene expression upon MnCl_2_ treatment in mutant larvae; however, we did not see alterations in the number of apoptotic cells as determined by TUNEL staining.

In summary, transcriptome analysis of *slc39a14^−/−^* zebrafish suggests that Mn overexposure affects a multitude of molecular processes. The future challenge will be the identification of the trigger event that leads to Mn-induced Ca^2+^ dyshomeostasis as well as mitochondrial and lysosomal dysfunction, a prerequisite for finding novel therapeutic targets for the treatment of Mn neurotoxicity.

### Mn toxicity in *slc39a14^−/−^* zebrafish causes impairment in retinal function

Transcriptome analysis revealed an unsuspected Mn toxicity effect in *slc39a14^−/−^* zebrafish, with more than 30 retinal phototransduction genes being differentially expressed. Changes in expression were accompanied by impaired VBA and an altered OKR. Combined with the reduced neuronal activity observed within the optic tectum, this suggests that Mn has toxic effects on the function of the zebrafish retina. Although this has not been observed in affected patients or rodent models of Mn overload, both Mn uptake transporters SLC39A8 and SLC39A14 are highly expressed in the retinal pigment epithelium (Leung et al., 2008). Furthermore, Mn plays an essential role in retinal function, in which it is required for normal ultrastructure of the retina (Gong and Amemiya, 1996). Possible differences between the human and zebrafish phenotypes might simply be caused by the direct contact of the zebrafish eye with Mn in the water, contributing to enhanced ocular Mn uptake and toxicity. We cannot exclude a direct effect of Mn on the oculomotor system and melanophore function leading to the changes in the OKR and VBA observed, but the large number of differentially expressed phototransduction genes, as well as reduced tectal neuronal activity, make Mn-induced retinal dysfunction more likely. It is plausible that Mn also affects non-retinal photoreceptors, which might link to the expression changes observed for several circadian clock genes, as well as the altered VMR. Mn administration in rats has previously been shown to cause dysregulation of circadian clock gene expressions (Li et al., 2017). Locomotor behavioural analysis of *slc39a14^−/−^* zebrafish did indeed reveal changes in the locomotor activity pattern, with decreased activity during the day and increased activity during the night, as well as an altered VMR at light-dark transitions, a behaviour linked to the function of non-visual photoreceptors (Fernandes et al., 2012).

### Loss of *slc39a14* function in zebrafish causes Mn deficiency

Perhaps the most intriguing observation was that most differentially expressed genes (235/266) in unexposed *slc39a14^−/−^* mutants normalised upon MnCl_2_ treatment. This indicates that although SLC39A14 deficiency leads to systemic Mn accumulation, it also causes deficiency of Mn in parts of the cell or specific types of cells due to its role as an Mn uptake transporter. This partial Mn deficiency might explain why chelation therapy in patients with HMNDYT2 is less effective compared to those with HMNDYT1, with some patients deteriorating upon Mn chelation (Tuschl et al., 2016; Marti-Sanchez et al., 2018; Rodan et al., 2018).

Mn deficiency in *slc39a14^−/−^* mutants suggests that some features of HMNDYT2 might overlap with those observed in SLC39A8 deficiency, an inherited Mn transporter defect leading to systemic Mn deficiency (OMIM 616721). Affected individuals present with intellectual disability, developmental delay, hypotonia, epilepsy, strabismus, cerebellar atrophy and short stature (Boycott et al., 2015; Park et al., 2015). However, HMNDYT2 does not share these features aside from cerebellar atrophy described in some patients.

As for Mn toxicity, the majority of ‘rescued’ genes map to the CNS. Several differentially expressed genes link to Ca^2+^ homeostasis and binding; however, these are different to those identified upon Mn overload. Notably, the expression of protocadherins and formin-related genes is reduced in unexposed *slc39a14^−/−^* mutants. Protocadherins are mainly expressed in the CNS where they are required for normal neural circuitry activity and regulate synaptic function (Kim et al., 2011). Loss of protocadherin function in mice has previously been associated with neurodegeneration (Hasegawa et al., 2016). Formins are required for the stabilisation of E-cadherins (Rao and Zaidel-Bar, 2016), which might link the changes observed in (proto-)cadherin expression with that of formin-associated genes.

How partial Mn deficiency arises within the brain of *slc39a14^−/−^* zebrafish remains to be determined. It might stem from differences in the expression patterns of various metal transporters. In the future, single-cell RNA sequencing, spatial transcriptomics and proteomics might allow us to distinguish neurons/glial cells affected by Mn neurotoxicity from those with deficiency. Identifying the overlap between chelator-treated and mutant larvae as well as analysis of Slc39a14-deficient neuronal cultures will aid to delineate the molecular events underlying partial Mn deficiency in *slc39a14^−/−^* mutants.

In conclusion, our results demonstrate that partial Mn deficiency might be an additional feature to Mn neurotoxicity in *slc39a14^−/−^* zebrafish. Overall, the *slc39a14^U801^* loss-of-function zebrafish mutants are an excellent disease model to study the disease pathogenesis of HMNDYT2 as well as Mn neurotoxicity.

## MATERIALS AND METHODS

### Zebrafish husbandry

Zebrafish were reared on a 14:10 h light-dark cycle at 28.5°C at the University College London (UCL) Zebrafish Facility. Embryos were obtained by natural spawning, and staging was performed according to standard criteria (Kimmel et al., 1995). Previously generated *slc39a14^U801^* loss-of-function zebrafish and their siblings were used for all experiments (Tuschl et al., 2016). Ethical approval for zebrafish experiments was obtained from the Home Office UK under the Animal Scientific Procedures Act 1986.

### Preparation of larvae for RNA and DNA extraction

The progeny of a single incross of *slc39a14^U801/+^* fish were raised in 10-cm Petri dishes filled with fish water (0.3 g/l Instant Ocean, 50 embryos per dish) at 28°C. At 2 dpf, half of the larvae were exposed to MnCl_2_ that was added to the fish water at a concentration of 50 µM (stock solution 1 M MnCl_2_, made up in water). After 72 h of exposure (at 5 dpf), single larvae were collected in the wells of a 96-well plate, immediately frozen on dry ice and stored at −80°C. For sequencing, frozen embryos were lysed in 100 µl RLT buffer (Qiagen) containing 1 µl of 14.3 M β-mercaptoethanol (Sigma). The lysate was allowed to bind to 1.8 volumes of Agencourt RNAClean XP (Beckman Coulter) beads for 10 mins. The plate was then applied to a plate magnet (Invitrogen) until the solution cleared and the supernatant was removed without disturbing the beads. While still on the magnet, the beads were washed three times with 70% ethanol and total nucleic acid was eluted from the beads as per the manufacturer's instructions. Nucleic acid samples were used for genotyping of individual larvae by KASP assay (LGC Genomics) according to the manufacturer's instructions, and the following primers were used: wild-type allele, 5′-GGCACATAATAATCCTCCATGGG-3′; mutant allele, 5′-GGGCACATAATAATCCTCCATGGT-3′; and common primer, 5′-CCCTGTATGTAGGCCTTCGGGTT-3′. After DNase treatment, RNA was quantified using either Qubit RNA HS assay or Quant-iT RNA assay (Invitrogen).

### Transcript counting

DeTCT libraries were generated as described previously (Collins et al., 2015). Briefly, 300 ng of RNA from each genotyped sample was fragmented and bound to streptavidin beads. The 3′ ends of the fragmented RNA were pulled down using a biotinylated polyT primer. An RNA oligo containing the partial Illumina adapter 2 was ligated to the 5′ end of the bound fragment. The RNA fragment was eluted and reverse transcribed using an anchored oligo-dT reverse-transcriptase primer containing one of the 96 unique index sequences and part of the Illumina adapter 1. The Illumina adapters were completed during a library amplification step and the libraries were quantified using either the BioPhotometer (Eppendorf) or Pherastar (BMG Labtech). This was followed by size selection for an insert size of 70-270 bases. Equal quantities of libraries for each experiment were pooled, quantified by qRT-PCR, and sequenced on either HiSeq 2000 or HiSeq 2500 (Illumina).

Sequencing data were analysed as described previously (Collins et al., 2015). Briefly, sequencing reads were processed with the DeTCT detag_fastq.pl (https://github.com/iansealy/DETCT) script and aligned to the GRCz11 zebrafish reference genome with BWA 0.5.10 (Li and Durbin, 2009). The resulting BAM files were processed using the DeTCT pipeline, which results in a list of regions (for simplicity referred to as genes in the Results) representing 3′ ends, together with a count for each sample. These counts were used for differential expression analysis with DESeq2 (Love et al., 2014). Each region was associated with Ensembl 95 (Yates et al., 2020) gene annotation based on the nearest transcript in the appropriate orientation. False positive 3′ ends representing, for example, polyA-rich regions of the genome, were filtered using the DeTCT filter_output.pl script with the ‘—strict’ option. Gene sets were analysed using the Cytoscape plugin ClueGO (Bindea et al., 2009) for GO enrichment and Ontologizer (Bauer et al., 2008) for ZFA Ontology enrichment.

### qRT-PCR

RNA extraction from 30 zebrafish larvae from the same genotype (homozygous mutant or wild-type) was performed using 500 µL TRIzol reagent (Invitrogen) according to the manufacturer's protocol and purified using the RNeasy MiniKit (Qiagen). cDNA was generated using GoScript Reverse Transcriptase (Promega). qRT-PCR was performed using GoTaq qPCR Master Mix (Promega) according to the recommended protocol. All samples were run in triplicates. qRT-PCR was carried out on a CFX96 Touch Real-Time PCR Detection System (Bio-Rad). Only primer pairs with R2 values >0.99 and amplification efficiencies between 95% and 105% were used. Relative quantification of gene expression was determined using the 2^−ΔΔCt^ method, with elongation factor 1α (*ef1α*, also known as *eef1a1l1*) as a reference gene (Livak and Schmittgen, 2001). The following primer sequences were used: *ef1a* forward, 5′-GTACTTCTCAGGCTGACTGTG-3′; *ef1a* reverse, 5′-ACGATCAGCTGTTTCACTCC-3′; *bdnf* forward, 5′-AGATCGGCTGGCGGTTTATA-3′; *bdnf* reverse 5′-CATTGTGTACACTATCTGCCCC-3′; *gnat2* forward, 5′-GCTGGCAGACGTCATCAAAA-3′; *gnat2* reverse, 5′-CTCGGTGGGAAGGTAGTCAG-3′; *hspa5* forward, 5′-GCTGGGCTGAATGTCATGAG-3′; *hspa5* reverse 5′-CAGCAGAGACACGTCAAAGG-3′; *opn1mw2* forward, 5′-GCTGTCATTTCTGCGTTCCT-3′; *opn1mw2* reverse, 5′-GACCATGCGTGTTACTTCCC-3′; *pde6ha* forward, 5′-CTCGCACCTTCAAGAGCAAG-3′; *pde6ha* reverse, 5′-CATGTCTCCAAACGCTTCCC-3′; *prph2b* forward, 5′-GCCCTGGTGTCCTACTATGG-3′; *prph2b* reverse, 5′-CTCTCGGGATTCTCTGGGTC-3′.

### ICP-MS analysis of metal ions

ICP-MS analysis of zebrafish larvae was performed as previously described (Tuschl et al., 2016). In brief, ten larvae of the same genotype, anaesthetised with MS-222 (4% Tricaine), were pooled and washed several times with distilled water. Samples were digested in 200 µl concentrated nitric acid at 95°C until dry and resuspended in 1* *mL 3% nitric acid. Further dilution with 20% nitric acid to a final volume of 2 ml was done prior to analysis. The metals (^24^Mg, ^44^Ca and ^55^Mn) were measured using an Agilent 7500ce ICP-MS instrument with collision cell (in He mode) and Integrated Autosampler (I-AS) using ^72^Ge as an internal standard. The following experimental parameters were used: (1) plasma: RF power, 1500* *W; sampling depth, 8.5 mm; carrier gas, 0.8* *l/min; make-up gas, 0.11* *l/min; and (2) quadrupole: mass range, 1-250 amu; dwell time, 100 msec; replicates, three; integration time, 0.1 s/point. Calibration solutions were prepared for each element between 0 and 200 ng/ml using certified reference standards (Fisher Scientific, UK).

### Apoptosis analysis

The TUNEL assay was used to determine apoptotic cell death. Larvae were fixed at 5 dpf overnight at 4°C in 4% paraformaldehyde (PFA) and 4% sucrose. Brains were manually dissected, transferred to methanol and stored at −20°C. After rehydration in PBS with 0.5% Triton X-100 (PBSTr), brains were permeabilised using 1× proteinase K for 15 min. Following washes in PBSTr, the samples were incubated at −20°C in pre-chilled ethanol:acetone (2:1) for 10 min, followed by washes in PBSTr. After a 1 h incubation in Apoptag equilibration buffer (Millipore), the samples were incubated in 35 µl of TdT enzyme mix [24 µl reaction buffer, 12 µl TdT enzyme (both Millipore), 1 µl 10% Triton X-100] at 37°C overnight. Following washes in PBSTr and incubation in blocking solution (for 1 ml: 100 μl normal goat serum, 10 μl of DMSO, 0.89 ml PBSTr) for 2 h at room temperature, the samples were incubated with polyclonal anti-digoxigenin-AP antibody (Roche) at a concentration of 1:2000 in blocking solution at 4°C overnight, and then developed using 4-nitro blue tetrazolium chloride and 5-bromo-4-chloro-3-indolyl-phosphate toluidine-salt (Roche). Imaging was performed in 80% methanol on a Nikon Eclipse E1000 microscope using the Openlab 4.0.2 software package.

### Whole mount *in situ* hybridisation chain reaction (HCR)

Larvae were fixed in PFA with 4% sucrose overnight at 4°C, transferred into PBS the next morning and the brain dissected by removing skin, cartilage and eyes with forceps. For each target mRNA, custom DNA probe sets were designed and single-stranded DNA (ssDNA) oligos ordered from Life Technologies, ThermoFisher. DNA HCR amplifiers (comprising a pair of fluorophore-labelled DNA hairpins for Alexa Fluor 488 and Alexa Fluor 568) and hybridisation, wash and amplification buffers were purchased from Molecular Instruments (molecularinstruments.org). *In situ* HCR was performed using a published protocol (https://files.molecularinstruments.com/MI-Protocol-HCRv3-Zebrafish-Rev7.pdf) (Choi et al., 2018). For probe sets and amplifier details for each target mRNA (*cfos* and *gad1b*), see Table S10. Imaging was performed on a Zeiss Z1 Lightsheet microscope with a 10× imaging objective. Whole-brain image stacks were registered to a *gad1b* reference brain aligned to Zebrafish Brain Browser (Marquart et al., 2015) co-ordinates using Advanced Normalization Tools (ANTs) as reported in Marquart et al. (2017).

Following registration, we applied image analysis using ImageJ, MATLAB and custom-written scripts in Python. We first applied a 3D median filter to the image stacks, and subsequently performed permutation testing to detect changes in *cfos* signal between two groups as described in Randlett et al. (2015). This resulted in image stacks with *cfos* pixels that were enhanced or suppressed between control and treatment groups. These image stacks were then processed to determine their distribution across 168 different anatomical regions. Publicly available masks for these anatomical regions were used (Gupta et al., 2018). We used a false discovery rate (FDR) threshold of 0.05%, resulting in a 99.5% significance threshold for each active voxel. Python script for active voxel calculation in each brain mask and the ANTs script for registration can be found on Figshare: https://dx.doi.org/10.6084/m9.figshare.19550998 and https://dx.doi.org/10.6084/m9.figshare.19551007.

### Locomotor behavioural analysis

The behavioural assay was conducted as described previously (Tuschl et al., 2016). In brief, zebrafish embryos and larvae were raised on a 14:10 h light/dark cycle at 28°C. Single larvae were transferred to each well of a flat-bottom, clear polystyrene 96-square-well plate (Whatman) in fish water (650 µl) at 4 dpf. Mn exposure was achieved by adding MnCl_2_ directly to the fish water at the desired concentration (stock solution of 1 M MnCl_2_ made in distilled water). The 96-well plate was maintained at a constant temperature (28.5°C) and exposed to a 14:10 h white light/dark schedule with constant infrared illumination within a custom-modified Zebrabox (Viewpoint Life Sciences). The locomotor behaviour of zebrafish larvae was tracked from 4 to 7 dpf using an automated video tracking system (Viewpoint Life Sciences). Larval movement was recorded using Videotrack Quantisation mode. The Videotrack detection parameters were empirically defined for clean detection of larval movement with minimal noise. A custom-designed MATLAB code was used to extract the average activity data of each larva as described previously (Rihel et al., 2010). Frame-by-frame analysis (25 frames per second) was performed as described by Ghosh and Rihel (2020) using the published MATLAB code.

### Optokinetic response

The optokinetic response (OKR) was examined using a custom-built rig to track horizontal eye movements in response to whole-field motion stimuli. Larvae at 4 dpf were immobilised in 1.5% agarose in a 35 mm Petri dish and analysed at 5 dpf. The agarose surrounding the eyes was removed to allow normal eye movements. Sinusoidal gratings with spatial frequencies of 0.05, 0.1, 0.13 and 0.16 cycles/degree were presented on a cylindrical diffusive screen that was 25 mm from the centre of the fish's head. Gratings had a constant velocity of 10 degrees/second and changed direction and/or spatial frequency every 20 s. Eye movements were tracked under infrared illumination (720 nm) at 60 Hz using a Flea3 USB machine vision camera and custom-written software. A custom-designed MATLAB code was used to determine the eye velocity in degrees per second (available at https://bitbucket.org/biancolab/okrsuite).

### Retinal histology

5 dpf larvae were fixed in 4% PFA overnight at 4°C. Dehydration was achieved by a series of increasing ethanol concentrations in PBS (50%, 70%, 80%, 90%, 95% and 100% ethanol). After dehydration, larvae were incubated in a 1:1 ethanol:Technovit 7100 solution (1% Hardener 1 in Technovit 7100 basic solution) for 1 h followed by incubation in 100% Technovit solution overnight at room temperature (Heraeus Kulzer, Germany). Larvae were than embedded in plastic moulds in Technovit 7100 polymerisation medium and dried at 37°C for 1 h. Sections of 3 μm thickness were prepared with a microtome, mounted onto glass slides and dried at 60°C. Sections were stained with Richardson (Romeis) solution (0.5% borax, 0.5% Azur II, 0.5% Methylene Blue) and slides were mounted with Entellan (Merck, Darmstadt, Germany). Images were taken in the brightfield mode of a BX61 microscope (Olympus).

### Experimental design and statistical analyses

Animals were divided into four experimental groups: unexposed homozygous *slc39a14^−/−^* mutants and their siblings (wild-type and heterozygous genotypes), and MnCl_2_-exposed homozygous *slc39a14^−/−^* mutants and their siblings (wild-type and heterozygous genotypes). For the DeTCT data, an equal number of wild-type and heterozygous embryos were selected (see Fig. 1 for numbers of embryos for each experimental group). This was to investigate the possibility of transcriptional changes in the heterozygous embryos compared with wild-type ones. This was not the case; the PCA showed that heterozygous embryos grouped with wild-type embryos and not separately. Differential expression analysis returned only 11 genes that were statistically different between untreated heterozygotes and untreated wild-type embryos (four of these genes are on the same chromosome as *slc39a14* and likely represent the effect of allele-specific expression linked to the mutation). There were only 20 genes that were differentially expressed between Mn-exposed heterozygotes and Mn-exposed wild-type embryos, with three being linked to *slc39a14*. These lists have been included in Table S1 for completeness. Because of the lack of effect, wild-type and heterozygous embryos were pooled as unaffected siblings for the remaining analyses.

Embryos were all derived from a single cross to minimise the amount of biological variance not caused by the experimental conditions (i.e. genotype and Mn exposure). One wild-type Mn-exposed embryo was excluded from the data after visual inspection of the PCA as it did not group with any of the other samples. DESeq2 was used for differential expression analysis with the following model: ∼genotype+treatment+genotype:treatment. This models the observed counts as a function of the genotype (homozygous versus siblings) and the treatment (Mn exposed versus unexposed) and an interaction between the two, and tests for significant parameters were performed using the Wald test with an adjusted *P*-value (Benjamini–Hochberg) threshold of 0.05.

The three groups of differentially expressed genes were defined as follows: (1) Mn toxicity, significant in Mn-exposed siblings versus unexposed siblings; (2) increased sensitivity, significant in Mn-exposed mutants versus unexposed siblings AND NOT significant in unexposed mutants versus unexposed siblings AND NOT significant in Mn-exposed siblings versus unexposed siblings; and (3) mutant effect, significant in unexposed mutants versus unexposed siblings. These groups were not mutually exclusive and some genes appeared in more than one group because of the way the groups were defined.

For qRT-PCR, metal and behavioural locomotor analysis, ANOVA with Tukey post hoc testing, and for OKR analysis, a two-tailed unpaired Student's *t*-test was used to determine statistical significance, using the GraphPad Prism software (version 5). For GO term analysis, the settings for ClueGO were as follows: a right-sided hypergeometric test (enrichment only) was used with the Bonferroni step-down (Holm–Bonferroni) correction for multiple testing, and terms with corrected *P*-values >0.05 were discarded. For ZFA enrichment analysis, the Ontologizer Parent-Child-Union calculation method was used with Bonferroni correction.

### Transcription factor motif analysis

Transcription factor motif enrichment was performed using HOMER's findMotifs.pl tool (v4.10.3) with default settings (Heinz et al., 2010). The GRCz11 promoter set used was created with HOMER's updatePromoters.pl tool based on RefSeq genes from −2000* *bp to 2000* *bp relative to the transcription start site.

## Supplementary Material

Supplementary information
