# Supplementary material for: Loss of slc39a14 causes simultaneous manganese hypersensitivity and deficiency in zebrafish
Source: Dis Model Mech. 2022 Jun 15;15(6):dmm044594. doi: 10.1242/dmm.044594 (PMC9227717; doi:10.1242/dmm.044594)
Supplement: Supplementary information [file dmm-15-044594-s1.pdf]

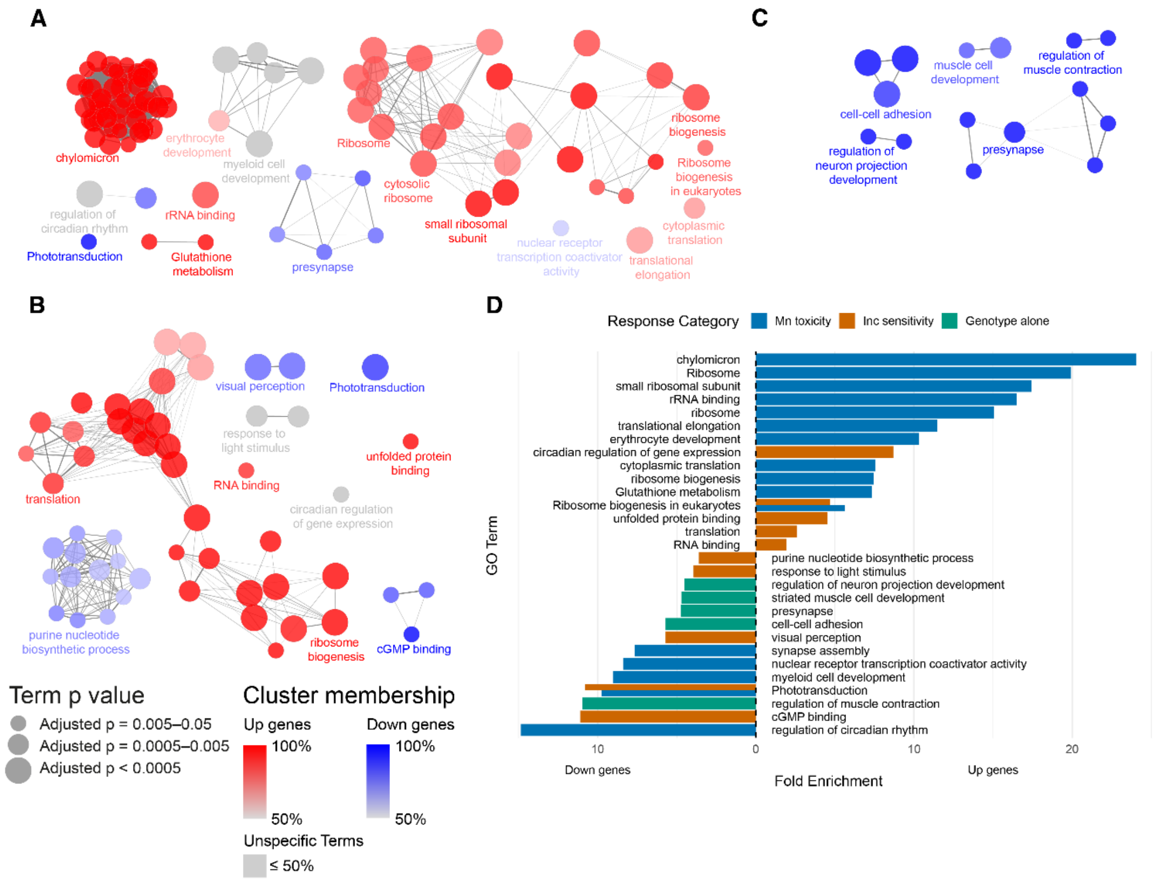

Fig. S1. Contribution to up- and down-regulated genes to GO enrichments.

(A-C) ClueGO network diagrams corresponding to the diagrams shown in Fig. 2C, 3G and 5E. Nodes represent enriched GO terms and edges connect nodes that share annotations to the significant genes. Each node is coloured according to the percentage of up/down-regulated genes contributing to the enrichment. Nodes coloured red have > 50% of the genes responsible for the enrichment upregulated, whereas blue nodes have > 50% of the genes downregulated. If the contribution of up/down-regulated genes is equal the nodes are grey. (A) Mn toxicity, (B) Increased sensitivity, (C) Mutant effect. (D) Bar chart showing the same information. The x-axis represents the Fold enrichment, [(genes in study set annotated to term / genes in study set) / (genes in reference set annotated to term / genes in reference set)]. Enrichment caused by upregulated genes are plotted to the right, whereas ones caused by downregulated genes are plotted to the left. blue = enrichments for the Mn toxicity set, orange = enrichments for the Increased sensitivity set, green = enrichments for the Mutant effect set.

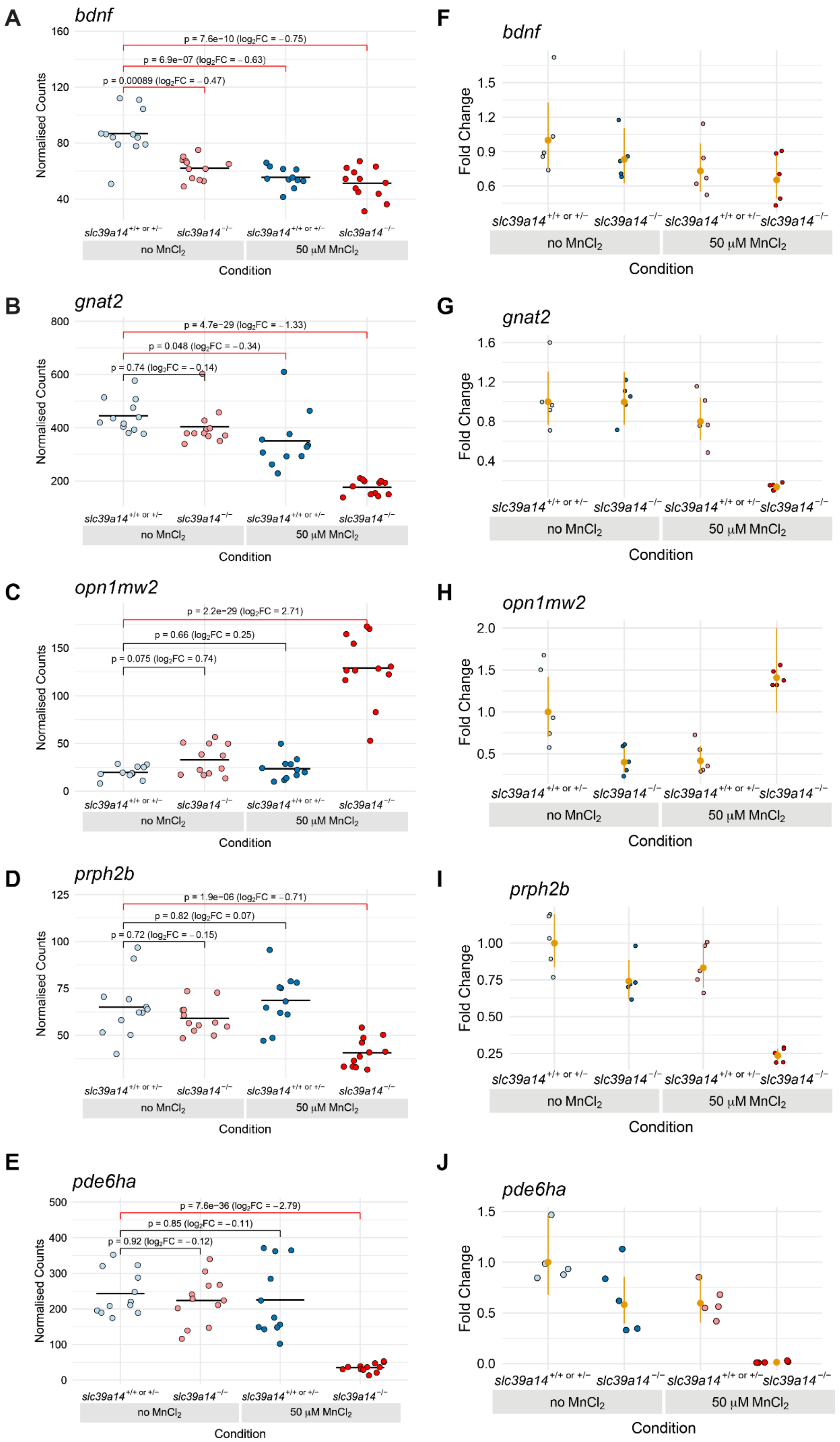
A
bdnf
p = 7.6e−10 (log2FC = −0.75)
p = 6.9e−07 (log2FC = −0.63)
p = 0.00089 (log2FC = −0.47)
Normalised Counts
B
gnat2
p = 4.7e−29 (log2FC = −1.33)
p = 0.048 (log2FC = −0.34)
p = 0.74 (log2FC = −0.14)
C
opn1mw2
p = 2.2e−29 (log2FC = 2.71)
p = 0.66 (log2FC = 0.25)
p = 0.075 (log2FC = 0.74)
D
prph2b
p = 1.9e−06 (log2FC = −0.71)
p = 0.82 (log2FC = 0.07)
p = 0.72 (log2FC = −0.15)
E
pde6ha
p = 7.6e−36 (log2FC = −2.79)
p = 0.85 (log2FC = −0.11)
p = 0.92 (log2FC = −0.12)
F
G
H
I
J
Fold Change
slc39a14+/+ or +/−
slc39a14−/−
no MnCl2
50 μM MnCl2
Condition

Fig. S2. qRT-PCR produces consistent results with transcriptome sequencing.

(A–D) Plots of the normalised counts for each sample for the genes *bdnf*, *gnat2*, *opn1mw2* and *prph2b*. Unexposed sibling embryos are light blue and $MnCl_2$ exposed ones are dark blue. Unexposed mutants are coloured light red and exposed mutants are dark red.

(E–H) Plots showing the qRT-PCR data for genes *bdnf*, *gnat2*, *opn1mw2* and *prph2b*. Values for individual samples are displayed as fold change relative to the mean value for unexposed siblings with the same colour scheme as in A–D. The mean and 95% confidence intervals for each condition are in orange.

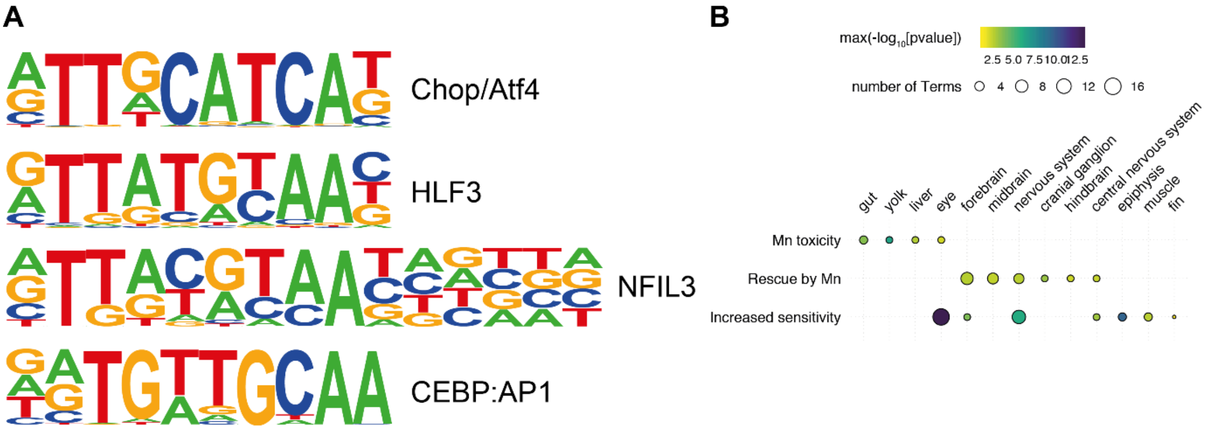

Fig. S3. Comparative analysis of gene sets.

(A) Example consensus binding motifs enriched in the promoters of genes that show increased sensitivity to Mn treatment in *slc39a14*$^{-/-}$ mutants (Group 1). The height of each base represents its frequency at that position in the consensus motif.

(B) Bubble plot of the ZFA enrichment results across the three categories of response. Individual enriched ZFA terms were aggregated to the tissue/organ level. For example, the terms optic cup, retina and photoreceptor cell are aggregated to the parent term eye. The size of each circle represents the number of individual terms enriched for the particular organ or tissue, and they are coloured by the smallest of the p values (-$\log_{10}$ scaled).

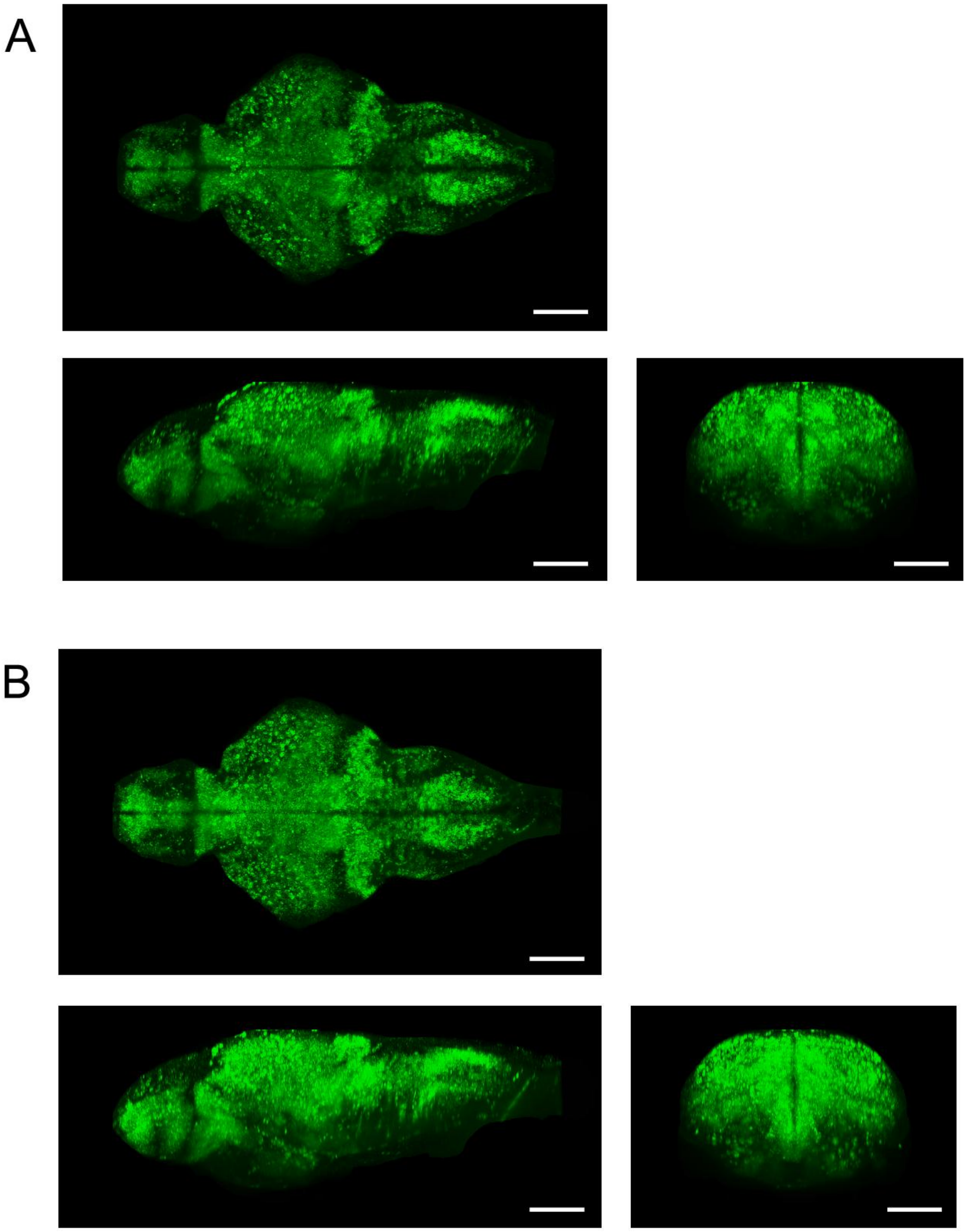

Fig. S4. *gad1b* expression is unchanged upon $MnCl_2$ exposure in *slc39a14*$^{-/-}$ zebrafish larvae. *gad1b* mRNA expression assessed by *in situ* HCR in the brain of (A) unexposed and (B) $MnCl_2$ (50µM) treated homozygous mutant larvae at 6 dpf. Scale bar 100µm.

Disease Models & Mechanisms • Supplementary information

Table S1. List of differentially expressed genes identified by DeTCT and grouped by Mn toxicity (differentially expressed in $MnCl_2$ exposed siblings compared with unexposed siblings), Increased sensitivity (differentially expressed in $MnCl_2$ exposed mutants compared with unexposed siblings, but not differentially expressed in unexposed mutants compared to unexposed siblings or exposed siblings compared with unexposed siblings) and Mutant effect (differentially expressed in unexposed mutants compared with unexposed siblings). Also included are genes differentially expressed in unexposed heterozygotes compared with unexposed wild-type embryos (het_noMnCl2_vs_wt_noMnCl2) and ones differentially expressed in exposed heterozygotes compared with exposed wild-type embryos (het_MnCl2_vs_wt_MnCl2). Genes highlighted in mustard are further discussed in the manuscript. Available at Figshare: https://dx.doi.org/10.6084/m9.figshare.19550899

Table S2. List of the 10 most highly up- and downregulated genes per group (Mn toxicity, Increased sensitivity and Mutant effect).

	gene	p-value	log2 fold change	GO term	
**Mn toxicity**					
**up**					
**1**	fads2	2.77E-07	1.80	fatty acid biosynthesis	
**2**	lonrf1l	2.02E-18	1.53	metal ion binding	
**3**	cry1bb	5.08E-14	1.44	circadian rhythm	
**4**	eevs	1.47E-10	1.38	lyase activity	
**5**	lonrf1	5.64E-16	1.25	metal ion binding	
**6**	ptgdsb.1	2.33E-21	1.24	prostaglandin	
**7**	apoa4b.2	4.37E-05	1.15	chylomicron	
**8**	fech	1.32E-11	1.11	erythrocyte development	
**9**	apoa4a	0.000111	1.09	chylomicron	
**10**	soul5	3.83E-10	1.00	erythrocyte development	
**down**					
**1**	nr1d4b	7.52E-16	-1.15	circadian rhythm	
**2**	dre-mir-132-2	2.3E-06	-1.15	miRNA	
**3**	dspa	9.43E-05	-0.99	cell-cell adhesion	
**4**	sv2a	8.77E-06	-0.95	synapse, transmembrane transport	
**5**	creb5b	5.95E-06	-0.93	transcription, metal ion binding	
**6**	sncb	3.86E-06	-0.85	dopaminergic	
**7**	TMEM151A	0.000181	-0.83	membrane	
**8**	nfil3-6	2.53E-05	-0.80	circadian rhythm	
**9**	large2	4.19E-05	-0.77	protein glycosylation	
**10**	cipca	1.86E-05	-0.76	circadian rhythm	
**Increased sensitivity**					**additive/synergistic**
**up**					
**1**	opn1mw2	1.07E-27	2.46	phototransduction	synergism
**2**	hspa5	1.91E-32	1.63	protein folding	synergism
**3**	faim2b	1.12E-13	1.60	autophagy	synergism
**4**	ptpdc1b	4.03E-06	1.60	protein dephosphorylation	additive
**5**	zwi	0.000101	1.53	myelin sheath	additive
**6**	fbxo21	0.005509	1.50	DNA binding	additive
**7**	cst14b.1	0.002159	1.43	endopeptidase inhibitor	additive
**8**	atf3	3.02E-05	1.38	transcription	additive
**9**	wbp2	1.36E-08	1.36	transcription	synergism
**10**	gtpbp1	3.26E-15	1.32	translational elongation	synergism
**down**					
**1**	ctsll	3.17E-15	-4.89	proteolysis	synergism

Disease Models & Mechanisms • Supplementary information

**2**	pde6ha	2.1E-35	-2.68	phototransduction	synergism
**3**	cyp11c1	3.55E-10	-2.39	oxidoreductase, metal ion/heme binding	synergism
**4**	pde6ha	2.91E-11	-2.09	phototransduction	synergism
**5**	slc1a2a	3.2E-19	-1.96	symporter, glutamate	synergism
**6**	dre-mir-124-4	0.000506	-1.76	miRNA	additive
**7**	grk1b	2.28E-10	-1.73	phototransduction	synergism
**8**	guca1g	2.1E-08	-1.71	phototransduction	synergism
**9**	six4a	0.000352	-1.69	transcription	additive
**10**	guca1d	1.17E-07	-1.68	phototransduction	synergism
**Mutant effect**					**Mn rescue**
**up**					
**1**	pxmp2	0.000741	1.17	peroxisomal membrane	not rescued
**2**	aacs	9.61E-07	1.16	fatty acid metabolism	not rescued
**3**	ddx1	0.000861	0.90	RNA helicase	rescued
**4**	mtmr12	0.000708	0.87	phosphatidylinositol dephosphorylation	rescued
**5**	sqstm1	0.000455	0.67	autophagy, metal ion binding	not rescued
**6**	alas1	7.52E-08	0.65	heme biosynthetic process	not rescued
**7**	dio3b	2.76E-05	0.62	thyroxine 5-deiodinase activity	not rescued
**8**	wdr44	0.000126	0.62	small GTPase binding	rescued
**9**	crebrf	0.000825	0.56	transcription, UPR	rescued
**10**	ces3	0.000236	0.55	hydrolase activity	rescued
**down**					
**1**	ebf3a	2.19E-06	-1.87	transcription, metal ion binding	rescued
**2**	klhl24b	0.000628	-1.62	glutamate receptor	rescued
**3**	pcdh7b	1.04E-05	-1.57	membrane, calcium ion binding	rescued
**4**	fam120c	4.07E-05	-1.55	nucleus	rescued
**5**	clec3ba	8.74E-05	-1.55	bone mineralization	rescued
**6**	ptprga	1.18E-06	-1.52	protein dephosphorylation	rescued
**7**	bmp7b	0.000316	-1.47	BMP signaling pathway	rescued
**8**	sv2a	8.11E-12	-1.46	synapse, transmembrane transport	rescued
**9**	phip	2.98E-05	-1.46	transcription	rescued
**10**	syt6a	4.21E-05	-1.46	synapse, calcium-ion regulated exocytosis	rescued

Disease Models & Mechanisms • Supplementary information

Table S3. List of the 10 most significantly differentially expressed genes with highest p-values for each group (Mn toxicity, Increased sensitivity and Mutant effect).

	**gene**	**p-value**	**log2 fold change**	**GO term**	
**Mn toxicity**					
**1**	sv2a	0.000864522	-0.95	Calcium, presynaptic neurotransmitter release	
**2**	igsf9ba	6.33E-05	-0.75	Cell adhesion, nervous system development	
**3**	sgcd	0.043419419	-0.68	Muscle	
**4**	CTBP1	0.01601469	-0.35	Transcription regulation	
**5**	dla	0.007417209	-0.42	Calcium, neurogenesis, notch	
**6**	parn	0.010107324	-0.37	Metal binding, mRNA degradation	
**7**	efnb2a	0.011782537	-0.36	Ephrin receptor binding, cell adhesion, axon guidance	
**8**	smurf2	0.008605029	-0.70	E3 ubiquitin-protein ligase, protein ubiquitination	
**9**	alas1	0.00084604	0.55	heme biosynthesis, response to hypoxia	
**10**	skib	0.000942987	-0.59	transcription factor, SMAD binding	
	gene	p-value	log2 fold change	GO term	
**Increased sensitivity**					additive/ synergistic
**1**	pde6ha	3.36859E-31	-2.68	Phototransduction	synergism
**2**	hspa5	1.53395E-28	1.63	Unfolded protein response	synergism
**3**	opn1mw2	5.72801E-24	2.46	Phototransduction	synergism
**4**	atp1a1b	1.27919E-16	0.95	Sodium/potassium-transport, metal binding	synergism
**5**	slc1a2a	7.34528E-16	-1.96	Excitatory amino acid transporter, glutamate reuptake	synergism
**6**	xbp1	7.34528E-16	0.84	Unfolded protein response	synergism
**7**	opn1mw1	1.91042E-14	-0.95	Phototransduction	synergism
**8**	rcvrna	3.81359E-14	-1.25	Phototransduction, calcium binding	synergism
**9**	slc1a2b	3.81359E-14	0.55	Excitatory amino acid transporter, glutamate reuptake	synergism
**10**	stm	6.39958E-13	0.63	Calcium ion transport, ephrin and notch receptor signalling	synergism
**Mutant effect**					Mn rescue
**1**	igf2bp2b	4.87E-14	-1.26	Translation regulation	rescued
**2**	gnai2a	2.96E-09	-0.70	G protein-coupled receptor signaling pathway, metal binding	rescued
**3**	anp32a	1.52E-08	-0.74	Apoptosis, RNA binding	rescued
**4**	sv2a	2.96E-08	-1.46	Calcium, presynaptic neurotransmitter release	rescued

Disease Models & Mechanisms • Supplementary information

**5**	adgrl1a	1.05E-07	-1.31	G protein-coupled receptor activity, cell adhesion	rescued
**6**	strn4	1.36E-07	-1.09	Calcium, calmodulin binding	rescued
**7**	igsf9ba	1.98E-07	-0.97	Cell adhesion, nervous system development	rescued
**8**	pcdh2aa15	5.7E-07	-0.68	Calcium ion binding, cell adhesion	rescued
**9**	ace2	1.58E-06	-0.88	Metal binding, angiotensin maturation	rescued
**10**	CU929544.1	1.58E-06	-0.48	Receptor-type tyrosine-protein phosphatase delta-like	rescued

Table S4. List of enriched Gene Ontology (GO) terms. Available at Figshare:

https://dx.doi.org/10.6084/m9.figshare.19550932

Table S5: Statistical analysis of qRT-PCR data. Available at Figshare:

https://dx.doi.org/10.6084/m9.figshare.19550938

Table S6: Enrichment of zebrafish anatomy (ZFA) terms. Available at Figshare:

https://dx.doi.org/10.6084/m9.figshare.19550959

Table S7. Locomotor activity data. Available at Figshare:

https://dx.doi.org/10.6084/m9.figshare.19550965

Table S8. HOMER enrichment analysis of transcription factor motifs. Available at Figshare:

https://dx.doi.org/10.6084/m9.figshare.19550968

Table S9. Optokinetic response data. Available at Figshare:

https://dx.doi.org/10.6084/m9.figshare.19550986

Table S10. HCR *in situ* hybridisation probes.

**Target mRNA**	***gad1b***
Amplifier	B1
Fluorophore	Alexa 488
Initiator I1	Initiator I2
gAggAgggCAgCAAACggAA	TAgAAgAgTCTTCCTTTACg
**Probe sequences**	
Pair1	Pair2
AACAGTGGGACATATCCCTTCTGTT	ACTGTAGTACCCGCCGTGGCATTCA
AAGCGCTCATTGTTGTCGCACGACA	TTGGAGAAATCTGTCTCATCGCGCG
CTATATGTCGTAAGGTGACTGTGTA	ACAAGCACCGGCTGTTATCCCAATG
GCGTGATTGGAGACCACCATTCGGA	AGGAAATCGATGTCCGACTTGGTGA
GCGGGCAGTAGATCTCGCGCGAACA	TGGATTGTGGGCTCCTCGCCGTTTT
AGAAAGGGGCTTTGTTAAAAGGGTG	GTTTACACACAGACCCCACTATTAC
TTTGGCCAGCCGATGATCTCCCGCA	GGTGAGAAGAGCGCATCTCCATCTC
CACGCACCATCCACATGTAACCACA	TTTCTGGACATCAACAGTCCTCCAC
AAATATTTATACCGCGCAACCATCA	GACATGCCTTTGGTTTTGACTTCAG
CAGACTGAATGGAAAAATCTGACAC	AGTCCACGGGAAACACCTCAATGCT
TTCTCTCTGACCAGGATGGCTGAAC	ATGGAGTTGCAGCCCTGCAGAATGC
**Target mRNA**	**cfos**
Amplifier	B3
Fluorophore	Alexa 546
Initiator I1	Initiator I2
gTCCCTgCCTCTATATCTTT	TTCCACTCAACTTTAACCCg
**Probe sequences**	
Pair1	Pair2
TGCACCGGGAAGACGCGTCGCAGTC	CGCTGTCGCCGCTCGGTGAAGCCGT
TGCTGCCCCTGCGATGCACTGATGT	AGGAGTCGGACGACTGATCGTTGCT
CACGTCGACGGTTGCGGCATTTCGC	TTTCAGCTTGCAGTGTATCGGTGAG
AGGTAGTGACGATCTCTGGGACTGA	ATGTGTTTGGTGTGGAAGAGACCAC
TGCAGATGGGTTTGTGTGCGGCGAG	CTGGGAAGCTGGCGTCGGCCGGGAT
CAGAGGAGATCATGGGCTGGACCAT	ATTGAGCTGCGCCGTTGGAGGGCGC
AGGCTGGAGTGCAGGTGACGACGGG	ACATGAAGGAAGACGTGTAGGTGGT
GCGCTAATATATCCAGAAAGTTAAA	CAAAAGTCGAAAAGCACGAGCTATC

Table S11. Download links for sequence data at the European Nucleotide Archive. Available at Figshare: https://dx.doi.org/10.6084/m9.figshare.19550989
